# Systems-level conservation of the proximal TCR signaling network of mice and humans

**DOI:** 10.1084/jem.20211295

**Published:** 2022-01-21

**Authors:** Philippe Nicolas, Jocelyn Ollier, Daiki Mori, Guillaume Voisinne, Javier Celis-Gutierrez, Claude Gregoire, Jeanne Perroteau, Régine Vivien, Mylène Camus, Odile Burlet-Schiltz, Anne Gonzalez de Peredo, Béatrice Clémenceau, Romain Roncagalli, Henri Vié, Bernard Malissen

**Affiliations:** 1 Centre d’Immunologie de Marseille-Luminy, Aix Marseille Université, Institut national de la santé et de la recherche médicale, Centre national de la recherche scientifique, Marseille, France; 2 Centre de Recherche en Cancérologie et Immunologie Nantes Angers, Institut national de la santé et de la recherche médicale, Centre national de la recherche scientifique, Université d'Angers, Université de Nantes, Nantes, France; 3 LabEx Immunotherapy–Graft–Oncology, Nantes, France; 4 Centre d’Immunophénomique, Aix Marseille Université, Institut national de la santé et de la recherche médicale, Centre national de la recherche scientifique, Marseille, France; 5 Institut de Pharmacologie et de Biologie Structurale, Université de Toulouse, Centre national de la recherche scientifique Université Paul Sabatier, Toulouse, France

## Abstract

We exploited traceable gene tagging in primary human T cells to establish the composition and dynamics of seven canonical TCR-induced protein signaling complexes (signalosomes) using affinity purification coupled with mass spectrometry (AP-MS). It unveiled how the LAT adaptor assembles higher-order molecular condensates and revealed that the proximal TCR-signaling network has a high degree of qualitative and quantitative conservation between human CD4^+^ and CD8^+^ T cells. Such systems-level conservation also extended across human and mouse T cells and unexpectedly encompassed protein–protein interaction stoichiometry. Independently of evolutionary considerations, our study suggests that a drug targeting the proximal TCR signaling network should behave similarly when applied to human and mouse T cells. However, considering that signaling differences likely exist between the distal TCR-signaling pathway of human and mouse, our fast-track AP-MS approach should be favored to determine the mechanism of action of drugs targeting human T cell activation. An opportunity is illustrated here using an inhibitor of the LCK protein tyrosine kinase as a proof-of-concept.

## Teaser

Nicolas et al. exploited traceable gene tagging in primary human T cells to establish at the systems level the composition and dynamics of TCR-induced signalosomes. It provides a decision-support tool accelerating definition of the mechanism of action of drugs targeting T cells.

## Introduction

TCR signaling is essential for both the development and function of T cells. When the TCR binds to an antigen, the immunoreceptor tyrosine-based activation motifs (ITAMs) found in the TCR-associated CD3 chains are phosphorylated by the protein tyrosine kinase (PTK) LCK. It allows the recruitment and activation of the cytosolic ZAP-70 PTK, which in turn phosphorylates tyrosine residues that are found in the cytoplasmic segment of transmembrane adaptors and serve as docking sites for the recruitment of cytosolic adaptors and enzymes. This results in the assembly of several discrete multiprotein signaling complexes known as signalosomes. They trigger multiple signaling pathways that ultimately impinge on cytoskeleton dynamics, metabolism, transcription, and translation (Balagopalan et al., 2010; Chakraborty and Weiss, 2014; Malissen et al., 2014; Sinclair et al., 2019), and their malfunction has pathologic consequences (Tangye et al., 2021).

We recently used affinity purification coupled with mass spectrometry (AP-MS) to analyze, at the systems level, the mouse signalosomes that assemble upon TCR engagement. It superseded coimmunoprecipitation approaches that address one protein–protein interaction at a time with limited quantitative insight and permitted us to define the composition and dynamics of the proteins (“prey") that assemble around 17 canonical proteins (“bait”) of the mouse TCR signal transduction network (Mori et al., 2021; Voisinne et al., 2019). Moreover, by combining the resulting interaction network (“interactome”) with the cellular abundance and interaction stoichiometry of the interacting proteins, we obtained a quantitative and contextual picture of each documented protein–protein interaction that helped rationalize the phenotypic effect of drug treatments intended to block T cell activation or of genetic mutations (Voisinne et al., 2019). Our original AP-MS study relied on gene-edited mice in which bait proteins were tagged with an affinity Twin Strep-tag (OST; Junttila et al., 2005). Although it permitted generation of primary T cells expressing physiologic levels of signalosomes amenable to AP-MS, our approach took up to a year to obtain the required mice cohorts. Accordingly, we recently developed a faster CRISPR/Cas9-based approach that renders ex vivo mouse primary T cells directly amenable to AP-MS analysis and permits establishing the composition and dynamics of signalosomes of interest in only 4 mo (Mori et al., 2021).

Although mouse models remain an integral part of therapeutic development and allow us to move from association studies to causality, numerous differences in immune signaling events have been reported between the mouse and human species, which likely affect therapeutic evaluation (Ernst and Carvunis, 2018; Pulendran and Davis, 2020; Wagar et al., 2018). Insights into the molecular bases of human immune receptor signaling have primarily relied on the analysis of transformed cell lines. In the case of T cells, the Jurkat human leukemic T cell line has provided invaluable information on TCR signaling (Chakraborty and Weiss, 2014) and has helped compare side by side the potency and redundancy of immune checkpoint inhibitors (Bhagwat et al., 2018; Celis-Gutierrez et al., 2019). However, most transformed human T cells lack key signaling proteins (Astoul et al., 2001), a feature that might preclude extending some of the conclusions of those studies to normal human T cells.

Here, we developed a fast-track platform for establishing, at the systems level, the composition and dynamics of the TCR-induced signalosomes that assemble in primary human T cells. Our CRISPR/Cas9-based approach used homology-directed repair (HDR) to insert the OST tag required for AP-MS at specific sites of the primary human T cell genome. It improved recent methods for editing primary T cells (Anderson et al., 2019; Chu et al., 2016; Gundry et al., 2016; Kornete et al., 2018; Roth et al., 2018; Seki and Rutz, 2018; Yao et al., 2018) in that it permits isolation by FACS of the low frequency of primary human T cells in which the OST tag required for AP-MS analysis was properly incorporated into the bait protein of interest. As a proof of concept, we used this platform to determine the composition, stoichiometry, and dynamics of the signalosomes assembling in primary human CD4^+^ and CD8^+^ T cells around ZAP-70, the linker for activation of T cells (LAT) transmembrane adaptor; the SLP-76 cytosolic adaptor; and the VAV1 guanine nucleotide exchange factor, before and after TCR engagement. Our study revealed that the proximal TCR signal transduction network has a high degree of qualitative and quantitative conservation across human CD4^+^ and CD8^+^ T cells and, more unexpectedly, across human and mouse T cells.

## Results

### Primary human CD4^+^ and CD8^+^ T cells amenable to fast-track interactomics

**Figure 1.**
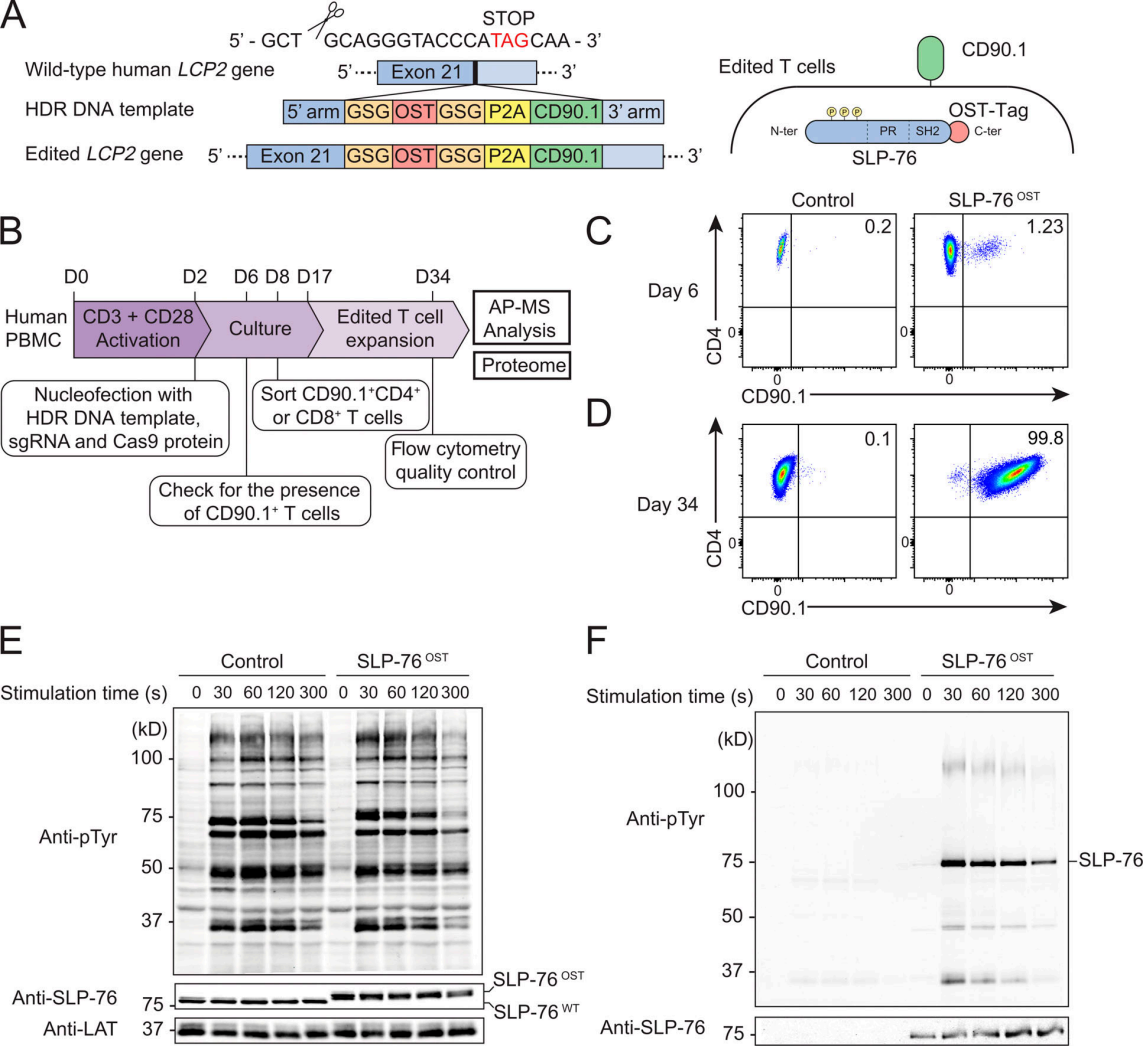
**Human primary CD4^+^ T cells amenable to fast-track AP-MS characterization of the SLP-76 signalosome. (A)** An sgRNA was designed to introduce a DSB 12 bp 5′ of the stop codon found in the human *LCP2* gene that codes for SLP-76 (Table S1). An 845-bp-long dsDNA was used as a template for HDR (Table S2). After HDR, CD4^+^ T cells are expected to coexpress intracytoplasmic SLP-76^OST^ molecules and the mouse CD90.1 protein at their surface. **(B)** Workflow used for editing, selecting, and expanding human primary CD4^+^ or CD8^+^ T cells expressing SLP-76^OST^ molecules amenable to AP-MS. **(C)** CD4^+^ T cells nucleofected with Cas9 alone (control) or Cas9-sgRNA RNP plus the HDR DNA template (SLP-76^OST^ edited) were analyzed 4 d after nucleofection by flow cytometry for CD90.1 expression. Data in C and D are representative of at least two experiments. **(D)** SLP-76^OST^–edited, CD90.1^+^CD4^+^ T cells were sorted, expanded in vitro, and analyzed for CD90.1 expression 1 d before AP-MS analysis, which corresponds to day 34 after the start of the culture. Also shown are control CD4^+^ T cells that were mock edited, sorted, and expanded in parallel. **(E)** Immunoblot analysis of equal amounts of proteins from total lysates of SLP-76^OST^–edited and control CD4^+^ T cells that were either left unstimulated (0 s) or stimulated for 30, 60, 120, or 300 s with anti-CD3 and anti-CD28 and probed with antibody to phosphorylated tyrosine (anti–p-Tyr), anti–SLP-76, or anti-LAT (loading control). **(F)** Immunoblot analysis of equal amounts of lysates of SLP-76^OST^–edited and control CD4^+^ T cells stimulated as in E and subjected to affinity purification on Strep-Tactin Sepharose beads, followed by elution of proteins with D-biotin, and probed with antibody to phosphorylated tyrosine (anti–p-Tyr) or anti–SLP-76. Left margin in E and F, molecular size in kilodaltons. Data in E and F are representative of at least two independent experiments.

We used the human *LCP2* gene that codes for SLP-76 to develop and validate a CRISPR/Cas9-based platform permitting rapid determination of the composition of signalosomes assembling in primary human T cells before and after TCR triggering. We adapted to primary human T cells an approach we recently developed for primary CD4^+^ T cells isolated from mice expressing a Cas9 endonuclease (Mori et al., 2021). It permits tracing, at the single-cell level, that the OST tag sequence required for AP-MS analysis is properly inserted at the 3′ end of at least one allele of the targeted human gene. Moreover, it allows FACS sorting of the low frequency of OST-edited primary human T cells before subjecting them to in vitro expansion and AP-MS analysis. Accordingly, we designed an sgRNA targeting the 3′ coding end of *LCP2* (Table S1), a double-stranded DNA (dsDNA) HDR template coding for an OST tag sequence, a self-cleaving peptide of the porcine teschovirus-1 2A (P2A), and for CD90.1 (also known as Thy1.1), a protein that is expressed at the surface of mouse T cells and is used here as a reporter (Fig. 1 A and Table S2). As a result of proper HDR, OST-tagged SLP76 (SLP-76^OST^) molecules remain expressed under the control of the endogenous *LCP2* gene, and the appended P2A cassette drives stoichiometric expression of CD90.1 reporter molecules (Fig. 1 A).

**Figure S1.**
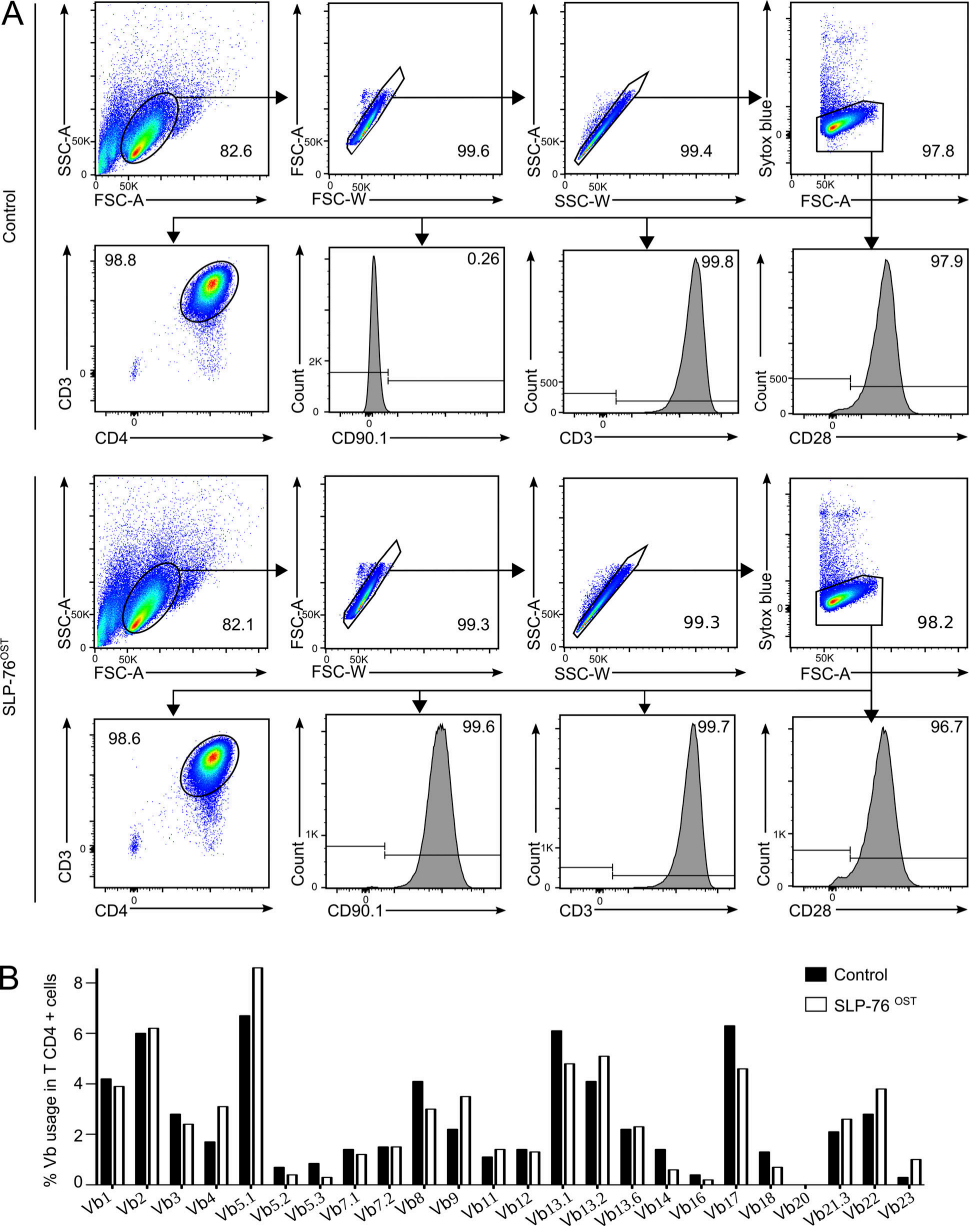
**Expression of CD4, CD90.1, CD3, and CD28 and TCR Vβ usage in SLP-76^OST^–edited and control CD4^+^ T cells. (A)** After gating on live singlets, SLP-76^OST^–edited and control CD4^+^ T cells that were expanded for 34 d were analyzed by flow cytometry for the expression of CD4, CD90.1, CD3, and CD28. Numbers in dot plots and histograms indicate the percentage of cells within the specified gates and the percentage of positive cells, respectively. Data are representative of three independent experiments. **(B)** SLP-76^OST^–edited and control CD4^+^ T cells were expanded for AP-MS and analyzed using a panel of 24 TCR Vβ–specific antibodies. The clonogram shows that both populations have preserved a rather similar polyclonal Vβ repertoire. Data are representative of two independent experiments. FSC, forward scatter; SSC, side scatter.

Primary human peripheral blood mononuclear cells (PBMCs) were activated for 2 d with anti-CD3 and anti-CD28 antibodies and nucleofected with both the HDR DNA template and Cas9-sgRNA ribonucleoprotein (RNP) complexes corresponding to the *LCP2* gene (Fig. 1 B). 4 d after nucleofection, 1.23% (range 0.25–7.5%; *n* = 5) of the T cells expressed CD90.1 (Fig. 1 C). CD90.1^+^CD4^+^ and CD90.1^+^CD8^+^ T cells were FACS sorted 6 d after nucleofection and expanded for 26 d (Vivien et al., 2018), to reach the substantial T cell numbers (1 × 10^8^ per time point) required for AP-MS. Activated PBMCs were also nucleofected with the vehicle alone and processed in parallel to serve as control cells. We first focused our analysis on CD90.1^+^CD4^+^ T cells and showed that at the end of expansion, they retained levels of CD3, CD4, and CD28 similar to those of expanded control cells (Figs. 1 D and S1 A). They also expressed a TCR Vβ repertoire that remained as diverse as that of expanded control CD4^+^ T cells (Fig. S1 B). Using MS analysis (Table S3), we showed that they contained the amino acid sequence expected to straddle the C-terminal end of the SLP-76 bait protein and the appended OST tag, therefore validating that HDR events occurred properly in the expanded CD4^+^ T cells coexpressing CD90.1. Immunoblot analysis further showed that SLP-76^OST^–edited and control CD4^+^ T cells expressed comparable levels of SLP-76, and that the presence of the OST sequence resulted in SLP-76 molecules with a higher molecular weight than that of control CD4^+^ T cells (Fig. 1 E), a result corroborating MS analysis of the SLP-76–OST junctional peptide sequence. Therefore, CD90.1 expression permits the tracing and sorting of primary human CD4^+^ T cells with a properly inserted OST tag.

The modification introduced in the *LCP2* gene was without measurable effects on the global pattern of TCR-induced tyrosine phosphorylation induced after stimulation with saturating concentrations of anti-CD3 plus anti-CD28 antibodies (Fig. 1 E). After lysis with the nonionic detergent *n*-dodecyl-β-D-maltoside, SLP-76^OST^ bait proteins were efficiently purified using Sepharose beads coupled to Strep-Tactin (Fig. 1 F). Purified SLP-76^OST^ bait proteins showed an increase in tyrosine phosphorylation that was maximal 30 s after TCR stimulation and led to their association with tyrosine phosphorylated species (Fig. 1 F). As expected, SLP-76 molecules were not recovered from control CD4^+^ T cells. Therefore, SLP-76^OST^ human CD4^+^ T cells can be used for AP-MS analysis of the SLP-76 signalosome.

### The SLP-76 interactome of human primary CD4^+^ T cells

**Figure S2.**
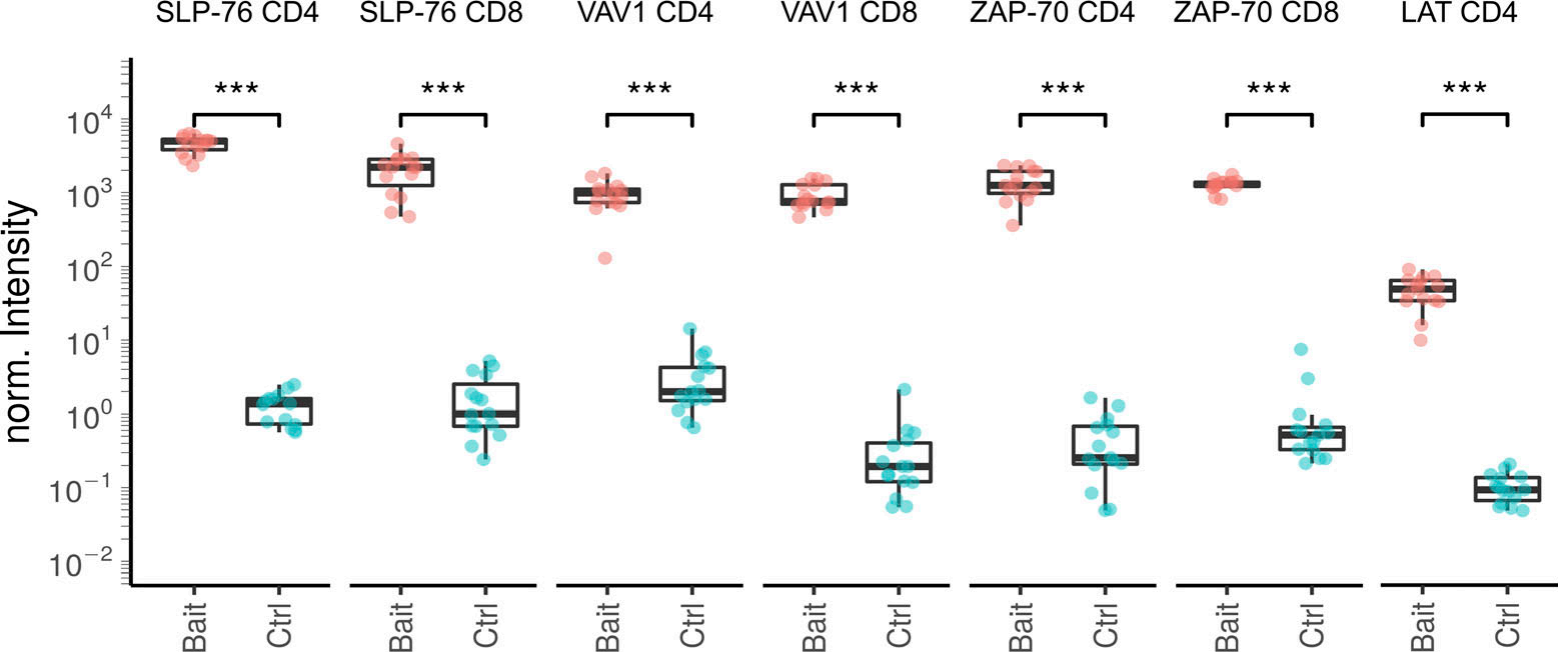
**Plot showing the reproducibility of the affinity purification and the enrichment of the bait protein.** Samples (Bait) correspond to LAT^OST^-edited CD4^+^ T cells, SLP-76^OST^–edited CD4^+^ and CD8^+^ T cells, VAV1^OST^-edited CD4^+^ and CD8^+^ T cells, and ZAP-70^OST^–edited CD4^+^ and CD8^+^ T cells as compared with the corresponding control samples (Ctrl). Protein intensities were normalized (norm.) according to the sample median intensity and averaged across technical replicates (see Materials and methods). Boxplot elements: Center line corresponds to median, box limits correspond to the first and third quartiles (Q1 and Q3), and whiskers indicate variability from Q1 − 1.5 × IQR to Q3 + 1.5 × IQR; IQR = Q3 − Q1, where IQR stands for interquartile range. Statistical significance was calculated using two-sided unpaired Welch’s *t*/Student’s *t* tests (***, P ≤ 0.001).

**Figure 2.**
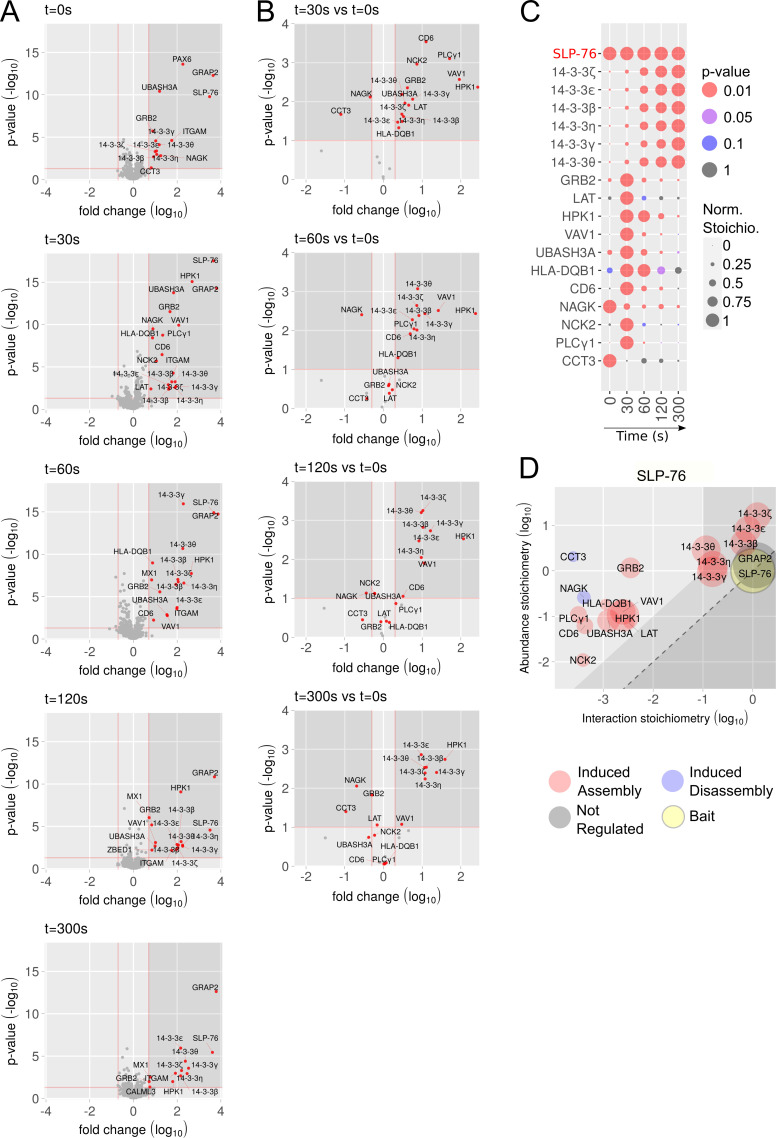
**Composition, dynamics, and stoichiometry of the SLP-76 interactome of human primary CD4^+^ T cells. (A)** Volcano plots showing proteins significantly enriched after affinity purification in CD4^+^ T cells expressing SLP-76^OST^ molecules compared with affinity purification in control CD4^+^ T cells expressing similar levels of untagged SLP-76 proteins before (*t* = 0 s) and 30, 60, 120, and 300 s after TCR-CD28 stimulation. In A and B, the x and y axes show the average fold-change (in log_10_ scale) in protein enrichment and the statistical significance, respectively. **(B)** Volcano plots showing proteins significantly enriched after affinity purification in SLP-76^OST^ CD4^+^ T cells 30, 60, 120, and 300 s after TCR-CD28 engagement compared with affinity purification in unstimulated SLP-76^OST^ CD4^+^ T cells. The x and y axes show the average fold-change (in log_10_ scale) in protein abundance and the statistical significance, respectively. **(C)** Dot plot showing the interaction stoichiometry over the course of TCR-CD28 stimulation of SLP-76 with its 17 high-confidence preys, the interaction stoichiometry of which changed following TCR stimulation. For each SLP-76–prey interaction, the interaction stoichiometry has been row-normalized to its maximum value observed over the course of TCR-CD28 stimulation (see normalized stoichiometry [Norm. Stoichio.] key). Also shown is the P value of the specified interactions (see P value key). The roles of the NAGK, CCT3, and HLA-DQB1 prey proteins that are found in the SLP-76 interactome of human CD4^+^ T cells remain to be elucidated. **(D)** Stoichiometry plot of the SLP-76 interactome. The SLP-76 bait is shown as a yellow dot, the preys that associate to SLP-76 after TCR-CD28 stimulation are specified by a red dot, and the preys that dissociate from SLP-76 after TCR-CD28 stimulation are specified by a blue dot. GRAP2, whose association to SLP-76 is constitutive, is also shown and specified by a gray dot. For each of the 17 TCR-regulated SLP-76–prey interactions, the ratio of prey to bait cellular abundance (abundance stoichiometry in log_10_ scale) was plotted as a function of the maximal interaction stoichiometry reached by the considered SLP-76–prey interaction over the course of TCR-CD28 stimulation (interaction stoichiometry in log_10_ scale). For instance, SLP-76 (893,261 copies per T cell; column I of the SLP76.CD4 tab in Data S1) is more abundant than LAT (62,934 copies per T cell), giving a ratio of prey to bait cellular abundance of −1.15 in log_10_ scale. Moreover, the maximal stoichiometry of the SLP-76–LAT interaction is reached at *t* = 30 s and corresponds to 0.0033 (−2.48 in log_10_ scale; column F of the SLP-76.CD4 tab in Data S1). Therefore, 2,954 (0.0033 × 893,261) molecules of SLP-76 are complexed to LAT at *t* = 30 s. As a result, 4.7% (2,947/62,934 × 100) of the available LAT proteins are complexed to SLP-76 30 s after TCR-CD28 engagement. The area including the SLP-76–prey interactions involving >10% of the available prey molecules is indicated in light gray and includes GRAP2 and six members of the 14-3-3 family (14-3-3 ζ, ε, β, η, γ, and θ). The limit imposed on interaction stoichiometries by the relative SLP-76–prey cellular abundances is shown by a dashed diagonal line that delimits a “forbidden” area (dark gray). Prey dot size is commensurate to its maximal protein enrichment over the course of stimulation.

Protein complexes assembling around the SLP-76^OST^ bait before or after cross-linkage of the TCR and CD28 molecules for 30, 60, 120, and 300 s were eluted with D-biotin and analyzed by liquid chromatography (LC) coupled to tandem MS (see Materials and methods). For each time point, three biological replicates were analyzed by MS. To distinguish truly interacting proteins from nonspecific contaminants, we compared our data with those of AP-MS experiments involving control CD4^+^ human T cells that were mock edited, sorted, and went through the same in vitro expansion protocol as SLP-76^OST^–edited CD4^+^ human T cells (Fig. S2). We identified 24 preys that showed >5-fold enrichment with a P value <0.05 in ≥1 of the 5 conditions of stimulation (Fig. 2 A and Data S1). As expected, the GRAP2 cytosolic adaptor (also known as GADS) constitutively associated to SLP-76. 17 prey proteins showed interaction stoichiometries that changed at least twofold with a P value <0.1 following anti-CD3 plus anti-CD28 stimulation, subsequently denoted as “TCR-regulated” (Fig. 2 B). Among those TCR-regulated preys, LAT, the cytosolic adaptors GRB2 and NCK2, the serine-threonine protein kinase hematopoietic progenitor kinase 1 (HPK1, also known as MEKKK1 or MAP4K1), VAV1, the ubiquitin-associated and SH3 domain-containing protein UBASH3A, the HLA-DQB1 MHC class II molecule, the transmembrane receptor CD6, and the phospholipase PLC-γ1 showed a transient pattern of binding to SLP-76 that peaked 30 s after stimulation (Fig. 2, B and C). In contrast, 6 TCR-regulated preys corresponding to the 14-3-3 ζ, ε, β, η, γ, and θ members of the 14-3-3 phosphoserine-phosphothreonine-binding protein family showed binding that increased from 60 to 300 s after activation, and 2 (NAGK and CCT3) dissociated from SLP-76 following TCR stimulation (Fig. 2, B and C).

By combining the interaction stoichiometries (Data S1) and the cellular abundances of the protein expressed in expanded human CD4^+^ T cells (Data S2), the SLP-76 signalosome was organized into a stoichiometry plot (Fig. 2 D; Voisinne et al., 2019). Accordingly, for each documented SLP-76–prey interaction, the ratio of prey to bait cellular abundance was plotted as a function of the maximal interaction stoichiometry reached by the considered bait–prey interaction over the course of TCR stimulation (Fig. 2 D). It showed, for instance, that SLP-76 molecules are 14-fold more abundant than LAT molecules and that 4.7% of the LAT molecules available in expanded human CD4^+^ T cells are complexed to SLP-76 30 s after TCR-CD28 engagement (see Fig. 2 D legend). Therefore, our integrated platform identified in 4 mo the composition, stoichiometry, and dynamics of the SLP-76 signalosome of primary human T cells before and after TCR engagement.

### Assessing the mechanisms of action of drugs in primary human T cells via AP-MS

**Figure 3.**
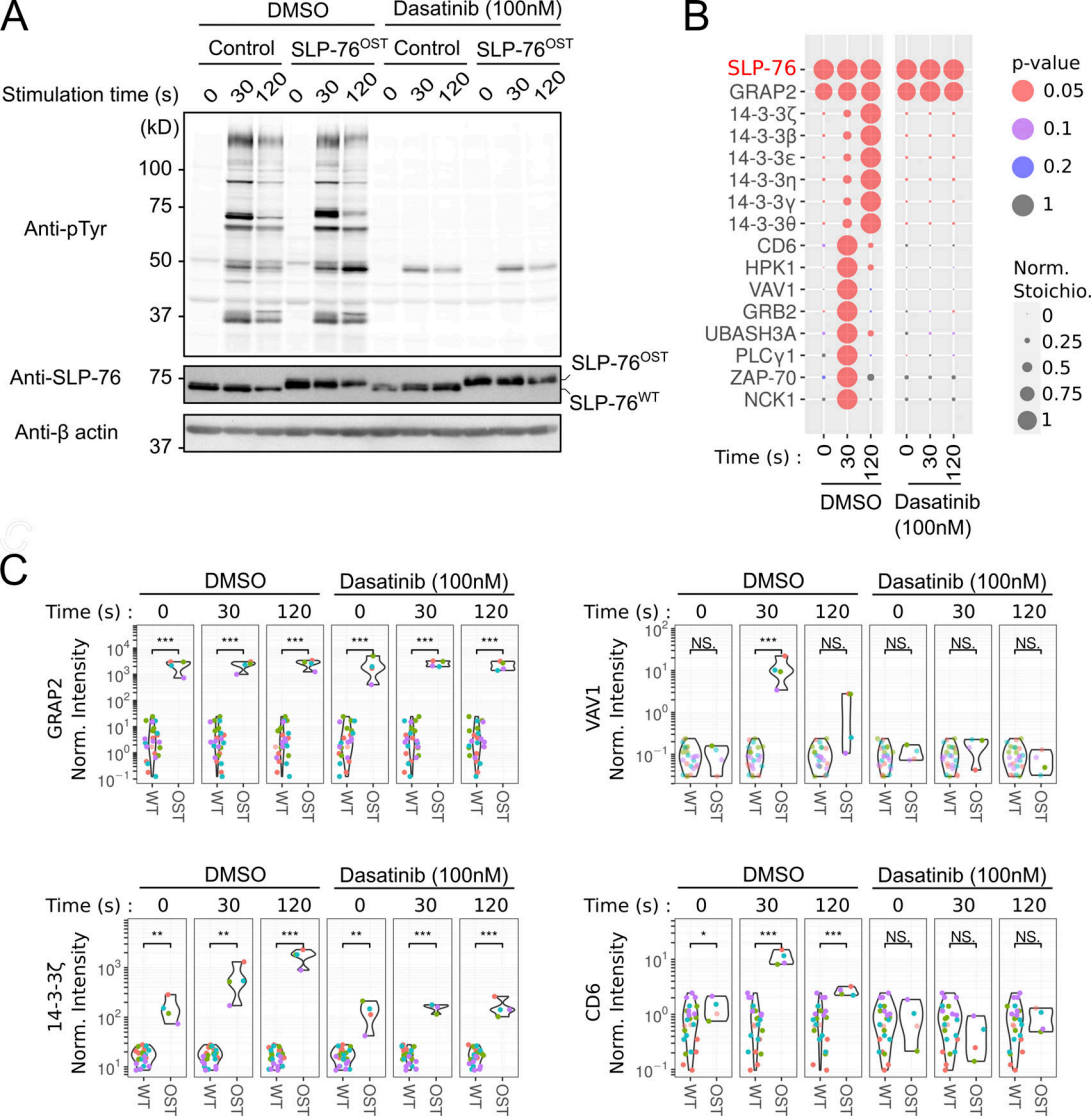
**Comparative analysis of the TCR-triggered SLP-76 interactomes of human primary CD4^+^ T cells in the presence or absence of dasatinib.** **(A)** Immunoblot analysis of equal amounts of proteins from total lysates of SLP-76^OST^ and control human CD4^+^ T cells that were expanded for AP-MS and either left unstimulated (0 s) or stimulated for 30 or 120 s with anti-CD3 and anti-CD28 and probed with antibody to phosphorylated tyrosine (anti–p-Tyr), anti–SLP-76, or anti-β actin (loading control). Before stimulation, T cells were incubated for 45 min at 37°C with either dasatinib (100 nM) or vehicle alone (DMSO) as specified. Dasatinib (100 nM) and DMSO were also kept during all the subsequent steps until cell lysis. Data are representative of at least two independent experiments. **(B)** Dot plot showing the interaction stoichiometry of SLP-76 with its high-confidence TCR regulated preys over the course of TCR-CD28 stimulation and in the presence of dasatinib or vehicle (DMSO). The stoichiometry of the constitutive SLP-76–GRAP2 interaction is also shown. For each SLP-76–prey interaction, the interaction stoichiometry has been row-normalized to its maximum value observed over the course of TCR-CD28 stimulation (see normalized stoichiometry [Norm. Stoichio.] key). Also shown is the P value corresponding to the enrichment in OST-tagged versus untagged AP-MS samples (see P value key). **(C)** Plot comparing the enrichment (shown as normalized intensity, see Materials and methods) of GRAP2, VAV1, 14-3-3 ζ, and CD6 in AP-MS samples from SLP-76^OST^ (OST) and control (WT) human CD4^+^ T cells over the course of TCR-CD28 stimulation in the presence of dasatinib or vehicle (DMSO). ***, P < 0.001; **, P < 0.01; *, P < 0.05; NS, P ≥ 0.05, where P corresponds to a Welch’s two-sided *t* test P value.

To determine whether our fast-track AP-MS platform can be used to assess the mechanisms of action of drugs targeting human T cell activation, we used dasatinib as a proof of concept. Dasatinib has been developed as an inhibitor of the BCR-ABL fusion protein (Tokarski et al., 2006). In addition, it blocks the adenosine triphosphate binding site of LCK (Blake et al., 2008) and is thus used to block T and chimeric antigen receptor (CAR) T cell activation (Mestermann et al., 2019). Upon TCR engagement, phosphorylation of CD3 ITAMs by LCK leads to the recruitment and activation of ZAP-70, which in turn phosphorylates tyrosine residues found in the SLP-76 adaptor (Chakraborty and Weiss, 2014). To assess whether the treatment of SLP-76^OST^–edited CD4^+^ human T cells with dasatinib prevented the assembly of the SLP-76 signalosome, SLP-76^OST^ and control CD4^+^ human T cells were subjected for AP-MS analysis as described above. Before stimulation with anti-CD3 plus anti-CD28, they were preincubated for 45 min at 37°C with either dasatinib (100 nM) or vehicle alone (DMSO). Dasatinib and DMSO were also kept during all the subsequent steps until cell lysis. Consistent with a previous study (Schade et al., 2008), immunoblot analysis showed that treatment with dasatinib but not with DMSO triggered a global decrease in TCR-induced protein tyrosine phosphorylation (Fig. 3 A). Consistent with these last results, dasatinib treatment totally prevented the assembly of the TCR-regulated SLP-76 interactome but preserved the constitutive SLP-76–GRAP2 interaction, which is independent of LCK activity (Fig. 3, B and C; and Data S3). Therefore, the possibility to render primary human T cells amenable to AP-MS permits us to precisely define whether a drug acts upstream or downstream of a given signalosome of the human TCR signal transduction network.

### Extending the AP-MS pipeline to the ZAP-70, LAT, and VAV1 molecules and to primary human CD8^+^ T cells

**Figure 4.**
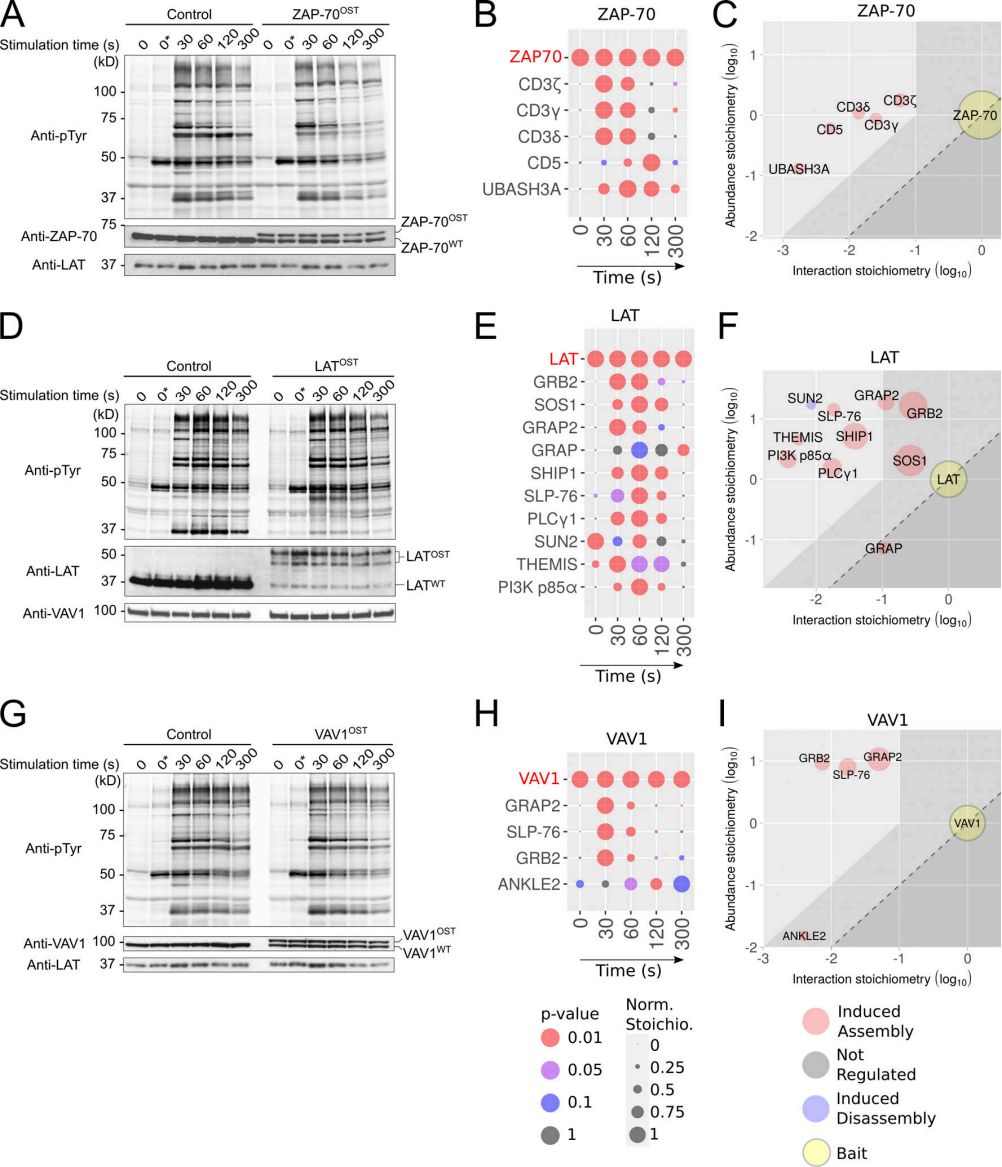
**Composition, dynamics, and stoichiometry of the ZAP-70, LAT, and VAV1 interactomes of human primary CD4^+^ T cells. (A)** Immunoblot analysis of equal amounts of proteins from total lysates of ZAP-70^OST^–edited and control CD4^+^ T cells that were expanded for AP-MS and either left unstimulated (0 s) or stimulated for 30, 60, 120, or 300 s with anti-CD3 and anti-CD28 and probed with antibody to phosphorylated tyrosine (anti–p-Tyr), anti–ZAP-70, or anti-LAT (loading control). Note that our traceable genetic edition of primary human T cells ensures that at least one allele of the targeted gene is edited. In the case of the *LCP2* gene, the editing process was highly efficient, and the two available LCP2 alleles incorporated the OST tag (Fig. 1 E). In contrast, in the case of the *ZAP70*, *LAT*, and *VAV1* genes, only one of the two alleles incorporated the OST tag, leaving the other allele in a WT configuration (Fig. 4, A, D, and G). Note that it has no impact on the determination of bait–prey interaction stoichiometry. **(B)** Dot plot showing the interaction stoichiometry over the course of TCR stimulation of the ZAP-70^OST^ bait with its five high-confidence preys, the interaction stoichiometry of which changed following TCR engagement. For B, E, and H, see description in Fig. 2 C. **(C)** Stoichiometry plot of the ZAP70^OST^ interactome. For C, F, and I, see description in Fig. 2 D. **(D)** Immunoblot analysis of equal amounts of proteins from total lysates of LAT^OST^-edited and control CD4^+^ T cells that were expanded for AP-MS and either left unstimulated (0 s) or stimulated for 30, 60, 120, or 300 s with anti-CD3 and anti-CD28 and probed with antibody to phosphorylated tyrosine (anti–p-Tyr), anti-LAT, or anti-VAV1 (loading control). **(E)** Dot plot showing the interaction stoichiometry over the course of TCR stimulation of LAT^OST^ with its 10 high-confidence preys, the interaction stoichiometry of which changed following TCR engagement. **(F)** Stoichiometry plot of the LAT^OST^ interactome. **(G)** Immunoblot analysis of equal amounts of proteins from total lysates of expanded VAV1^OST^-edited and control CD4^+^ T cells that were either left unstimulated (0 s) or stimulated for 30, 60, 120, or 300 s with anti-CD3 and anti-CD28 and probed with antibody to phosphorylated tyrosine (anti–p-Tyr), anti-VAV1, or anti-LAT (loading control). **(H)** Dot plot showing the interaction stoichiometry over the course of TCR stimulation of VAV1^OST^ with its four high-confidence preys, the interaction stoichiometry of which changed following TCR engagement. **(I)** Stoichiometry plot of the VAV1^OST^ interactome. Data in A, D, and G are representative of at least two independent experiments. Left margin in A, D, and G, molecular size in kilodaltons. To avoid cropping the immunoblots shown in A, D, and G, we kept an additional condition denoted *t* = 0 * s, in which T cells were incubated for 15 min on ice with anti-CD3 and anti-CD28 antibodies and incubated at 37°C for 5 min before lysis. In contrast, in the “regular” *t* = 0 s condition, T cells were incubated without anti-CD3 and anti-CD28 antibodies for 15 min on ice and incubated at 37°C for 5 min before lysis. Both conditions resulted in a similar pattern of tyrosine phosphorylated proteins.

**Figure 5.**
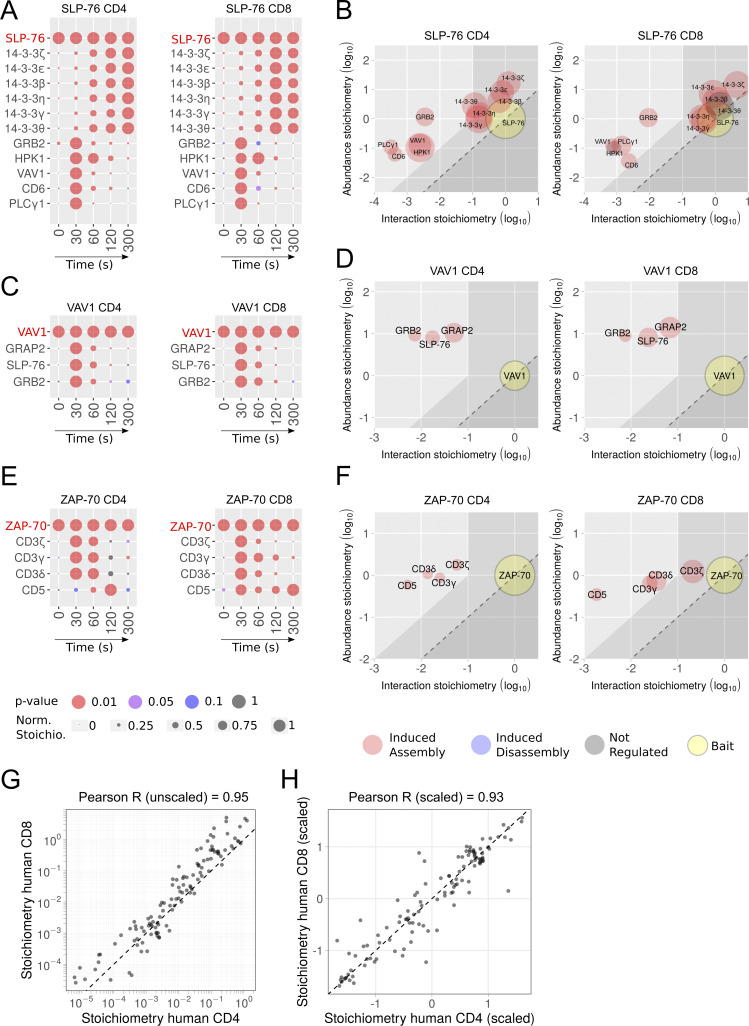
**Comparative analysis of the SLP-76, VAV1, and ZAP-70 interactomes of human primary CD4^+^ and CD8^+^ T cells. (A)** Dot plots showing the interaction stoichiometry of SLP-76 in human CD4^+^ and CD8^+^ T cells over the course of TCR-CD28 stimulation. In A–F, only the preys shared between CD4^+^ and CD8^+^ T cells are shown. For A, C, and E, see description in Fig. 2 C. **(B)** Stoichiometry plots of the SLP-76 interactome of human CD4^+^ and CD8^+^ T cells. For B, D, and F, see description in Fig. 2 D. **(C)** Dot plots showing the interaction stoichiometry of VAV-1 in human CD4^+^ and CD8^+^ T cells over the course of TCR-CD28 stimulation. **(D)** Stoichiometry plots of the VAV1 interactome of human CD4^+^ and CD8^+^ primary T cells. **(E)** Dot plots showing the interaction stoichiometry of ZAP-70 in human CD4^+^ and CD8^+^ T cells over the course of TCR-CD28 stimulation. The ZAP-70–CD3ε interaction occurring in CD8^+^ T cells is of high confidence (47-fold enrichment with a P value <0.05 at *t* = 30 s; Data S1). After slightly relaxing the cutoff values corresponding to ZAP-70–CD3ε interaction occurring in CD4^+^ T cells (4.3-fold enrichment with a P value <0.05 at *t* = 30 s; Data S1), a sixth interactor corresponding to CD3ε can also be added to the list of ZAP-70 interactors shared between mice and humans. **(F)** Stoichiometry plots of the ZAP-70 interactomes of human CD4^+^ and CD8^+^ T cells. **(G)** The interaction stoichiometries of TCR-regulated interactions identified in both human CD4^+^ and CD8^+^ T cells and corresponding to the SLP-76, VAV1, and ZAP-70 baits were compared across all conditions of stimulation. **(H)** Same comparison as in G using scaled (Z-score) interaction stoichiometries to compare variations of interaction stoichiometries relative to their means. Also shown in G and H are the Pearson correlation coefficient and a dashed line corresponding to equal stoichiometries (G) or scaled stoichiometries (H). Scaling was performed for each interaction by subtracting the mean and dividing it by the SD of log-transformed stoichiometries across all conditions of stimulation.

Next, we applied our approach to the ZAP-70, LAT, and VAV1 molecules of human CD4^+^ T cells (Fig. 4) and to the ZAP-70, SLP-76, and VAV1 molecules of human CD8^+^ T cells (Fig. 5). Primary human CD4^+^ and CD8^+^ T cells were activated, nucleofected, expanded, and subjected to quality control as described for SLP-76^OST^ CD4^+^ human T cells (see Table S1 and Table S2 for the sequences of the corresponding sgRNA and HDR templates). The appended OST had no detectable effect on the global pattern of TCR-induced tyrosine phosphorylation of human CD4^+^ T cells that were edited to express ZAP-70^OST^, LAT^OST^, and VAV1^OST^ bait proteins and the cells were thus subsequently expanded for 26 d (Fig. 4, A, D, and G). The sequences expected to straddle the C-terminal end of the ZAP-70, LAT, and VAV1 molecules and the appended OST tag were identified using MS analysis (Table S3), permitting efficient enrichment of the corresponding baits with Strep-Tactin (Fig. S2). The protein complexes assembling around each of the baits were analyzed by AP-MS as described for the SLP-76^OST^ bait (Fig. 4, B, E, and H; and Data S1).

The ZAP-70 interactome contained 13 high-confidence preys (Data S1), among which 5 corresponding to the CD3γ, CD3δ, and CD3ζ (also known as CD247) subunits of the TCR-CD3 complex, the transmembrane receptor CD5, and UBASH3A showed a TCR-regulated and transient pattern of binding to ZAP-70 (Fig. 4 B). After slightly relaxing the cutoff values, the CD3ε subunit of the TCR-CD3 complex (4.3-fold enrichment with a P value <0.05 at *t* = 30 s; Data S1) was also identified among the TCR-regulated ZAP-70 interactors. The LAT interactome contained 10 TCR-regulated preys that showed a pattern of binding that peaked 30–60 s after TCR stimulation (Fig. 4 E), and its composition was consistent with that obtained by addressing one interactor at a time (Balagopalan et al., 2015). The VAV1 interactome contained five high-confidence preys (Data S1), three of which, corresponding to GRB2, GRAP2, and SLP-76, showed a transient pattern of binding that peaked 30 s after TCR stimulation (Fig. 4 H). In the case of human CD8^+^ T cells, the ZAP-70^OST^, SLP-76^OST^, and VAV1^OST^ baits were efficiently purified with Strep-Tactin (Fig. S2), their C-terminal sequences were validated by MS (Table S3), and the protein complexes they assembled upon TCR plus CD28 cross-linkage were identified by AP-MS (Fig. 5 and Data S1). Therefore, our approach is readily scalable and can be extended to human CD8^+^ T cells.

### Comparison of the proximal TCR signaling network of primary human CD4^+^ and CD8^+^ T cells

Our AP-MS datasets offer the unprecedented possibility to compare the composition, dynamics, and stoichiometry of the ZAP-70, SLP-76, and VAV1 signalosomes that assemble in both human CD4^+^ and CD8^+^ T cells before and during 300 s of TCR engagement. The composition of the TCR-regulated SLP-76 interactome of human CD4^+^ and CD8^+^ T cells extensively overlapped (Fig. 5 and Data S1). Among the 17 TCR-regulated SLP-76 interactors identified in human CD4^+^ T cells, 14-3-3 ζ, ε, β, η, γ, and θ and GRB2, HPK1, VAV1, CD6, and PLCγ1 preys were shared with human CD8^+^ T cells (Fig. 5, A and B; and Data S1). After slightly relaxing cutoff values, a 12th interactor corresponding to UBASH3A (4.8-fold enrichment with a P value <0.05 at *t* = 30 s) can be further added to the list of TCR-regulated SLP-76 interactors shared between human CD4^+^ and CD8^+^ T cells (Data S1). Moreover, these interactors showed similar binding kinetics in both CD4^+^ and CD8^+^ human T cells (Fig. 5 A). For instance, PLCγ1, VAV1, and GRB2 showed a transient pattern of binding to SLP-76 that peaked 30 s after TCR engagement.

CD6 is a transmembrane receptor important for strengthening the interaction between T cells and antigen-presenting cells (APCs) and for sustaining TCR-induced cell proliferation (Meddens et al., 2018). Consistent with our former AP-MS analysis demonstrating that in mouse CD4^+^ T cells, SLP-76 is part of the CD6 signalosome (Mori et al., 2021), CD6 transiently interacted with SLP-76 30 s after TCR engagement in both human CD4^+^ and CD8^+^ T cells (Fig. 5 A). ZAP-70 binds to phosphorylated CD3 ITAM (Chakraborty and Weiss, 2014), and the ZAP-70 interactomes of both human CD4^+^ and CD8^+^ T cells contained all the subunits of the CD3 complex (Fig. 5 E). Consistent with coimmunoprecipitation studies in human thymocytes (Gary-Gouy et al., 1997), the CD5 transmembrane receptor was also found in the TCR-regulated ZAP-70 interactome of both CD4^+^ and CD8^+^ human T cells. Therefore, the composition and dynamics of the proximal TCR signal transduction protein network are largely conserved between human CD4^+^ and CD8^+^ T cells.

### Dismantling the SLP-76 interactomes of primary human CD4^+^ and CD8^+^ T cells

In both human CD4^+^ and CD8^+^ T cells, HPK1 interacted with SLP-76 30 and 60 s after TCR engagement, leading to serine-threonine phosphorylation of SLP-76 and its subsequent interaction with six 14-3-3 family members (14-3-3 ζ, ε, β, η, γ, and θ; Fig. 5 A). These last interactions were of high stoichiometry (Fig. 5 B) and reached a plateau 120 s after stimulation. They contributed to a HPK1-triggered negative feedback loop, leading to the subsequent displacement of the interaction of SLP-76 with PLCγ1, VAV1, and GRB2 (Fig. 5 A) and, in turn, to TCR signal termination (Di Bartolo et al., 2007; Lasserre et al., 2011; Yablonski, 2019).

**Figure S3.**
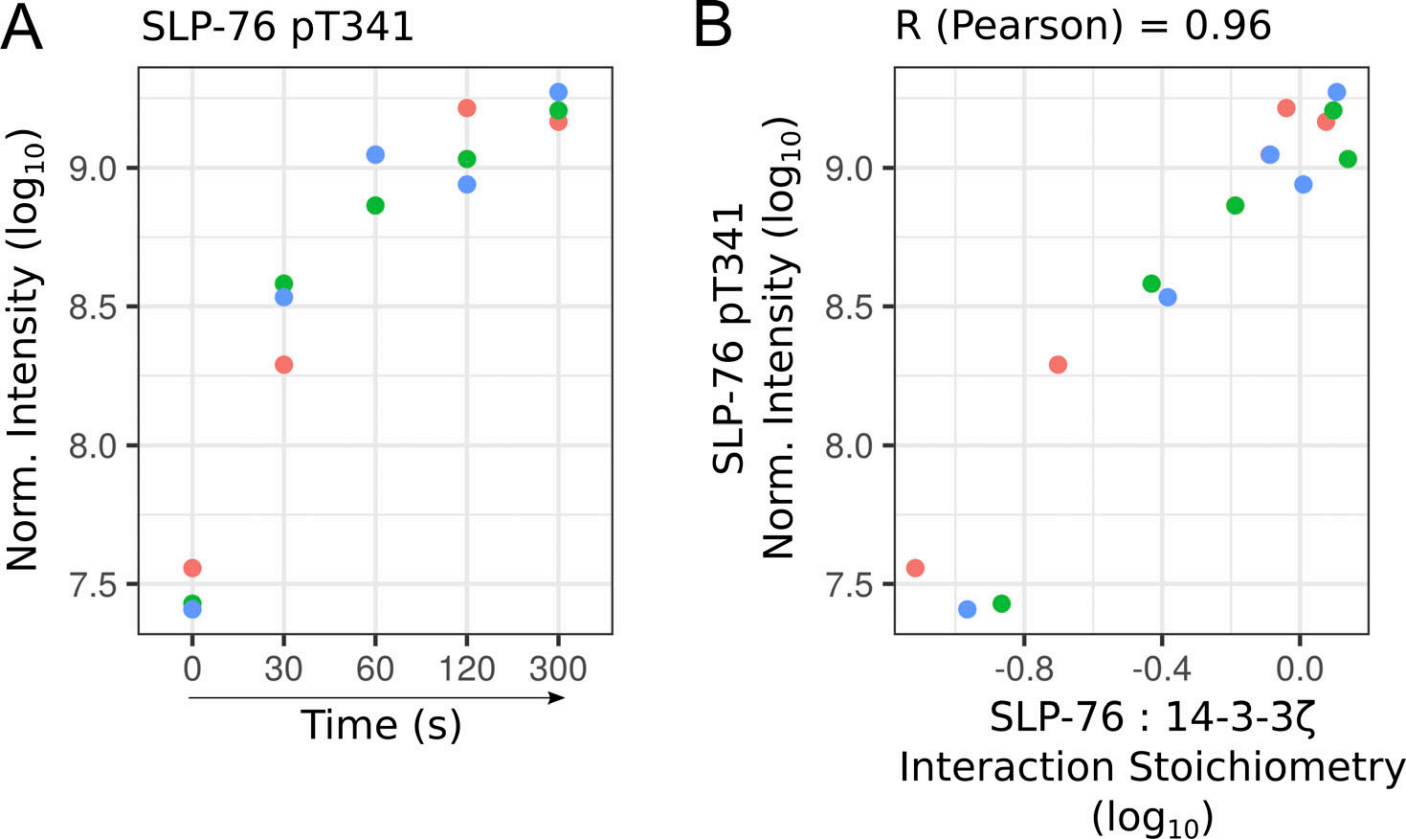
**Phosphorylation dynamics of the threonine residue found at position 341 of SLP-76. (A)** Plot showing the log_10_-transformed intensity for the phospho-site corresponding to the threonine residue found at position 341 of SLP-76 (SLP-76-pT341) over the course of TCR stimulation. Phosphorylated peptide intensity was quantified using AP-MS samples of SLP-76^OST^ human CD4^+^ T cells. Data corresponding to each of the three biological replicates are specified by dots of a distinct color. Norm., normalized. **(B)** Plot showing the correlation between SLP-76-pT341 intensity and the stoichiometry of the interaction between SLP-76 and its high-confidence prey 14-3-3ζ, both quantified from AP-MS data. Our dataset did not permit identification of additional phosphorylated peptides with high confidence in the SLP-76 bait and the other baits.

When HPK1 is recruited into the SLP-76 signalosome, it induces the phosphorylation of the serine residue found at position 376 of SLP-76 (Di Bartolo et al., 2007). However, in stark contrast to HPK1-deficient mice, TCR-induced proliferation was not enhanced in mice in which serine 376 of SLP-76 has been replaced by an alanine (Navas et al., 2017), suggesting that additional SLP-76 serine/threonine residues are phosphorylated and contribute to 14-3-3 protein recruitment. Along this line, we noted that the phosphorylation of the threonine residue found at position 341 of SLP-76 was induced by TCR stimulation (Fig. S3 A). Moreover, its phosphorylation dynamics correlated with the extent of interaction between SLP-76 and the 14-3-3 preys (a point illustrated in Fig. S3 B using the 14-3-3 ζ prey). Therefore, once phosphorylated, threonine 341 of SLP-76 might contribute to the recruitment of 14-3-3 family members, as postulated by a previous bioinformatics study (Madeira et al., 2015).

### The stoichiometries of interactions established by SLP-76, ZAP-70, and VAV1 are conserved in human CD4^+^ and CD8^+^ T cells

**Figure S4.**
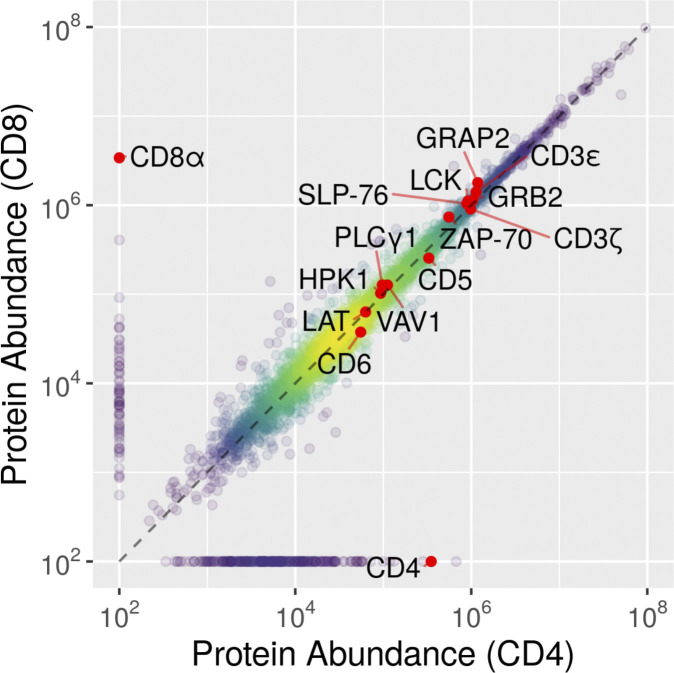
**Comparison of the cellular abundance of the proteins quantified in CD4^+^ and CD8^+^ primary human T cells.** Using the “proteomic ruler” method (Wiśniewski et al., 2014), we were able to quantify protein abundance for 5,151 and 4,905 protein groups in expanded CD4^+^ and CD8^+^ primary human T cells, respectively. 8.6% of the quantified proteins were expressed only in CD4^+^ or CD8^+^ T cells. A few proteins relevant for the present study are highlighted.

Analysis of the proteome of expanded human CD4^+^ and CD8^+^ T cells showed that 91% of the detectable proteins were expressed in both CD4^+^ and CD8^+^ T cells, of which >95% showed <2-fold difference in abundance (Fig. S4 and Data S1). As a result, each of the 11 TCR-regulated preys shared between the SLP-76 interactome of human CD4^+^ and CD8^+^ T cells occupied a similar position on the y axis stoichiometry plots (Fig. 5 B). Likewise, each of them occupied a similar position on the x axis of stoichiometry plots, demonstrating the existence of a high degree of interaction stoichiometry conservation between the SLP-76 interactome of human CD4^+^ and CD8^+^ T cells (Fig. 5 B, Data S1, and Data S2). A trend toward interaction stoichiometry conservation was also observed across human CD4^+^ and CD8^+^ T cells for VAV1 and ZAP-70 (Fig. 5, D and F). As a result, comparison of the interaction stoichiometries of all the bait–prey interaction involving ZAP-70, SLP-76, and VAV1 and detected in both human CD4^+^ and CD8^+^ T cells showed a Pearson correlation coefficient of 0.95 (Fig. 5 G). In addition, scaled (Z-score) interaction stoichiometries were highly correlated (Pearson correlation coefficient of 0.93), indicating that interaction kinetics were also conserved between human CD4^+^ and CD8^+^ T cells (Fig. 5 H). Therefore, the composition, kinetics of assembly–disassembly, and interaction stoichiometry of the ZAP-70, SLP-76, and VAV1 signalosomes of the proximal TCR signal transduction network are globally conserved between human CD4^+^ and CD8^+^ T cells.

### Comparison of the proximal TCR signaling network of human and mouse CD4^+^ T cells

**Figure 6.**
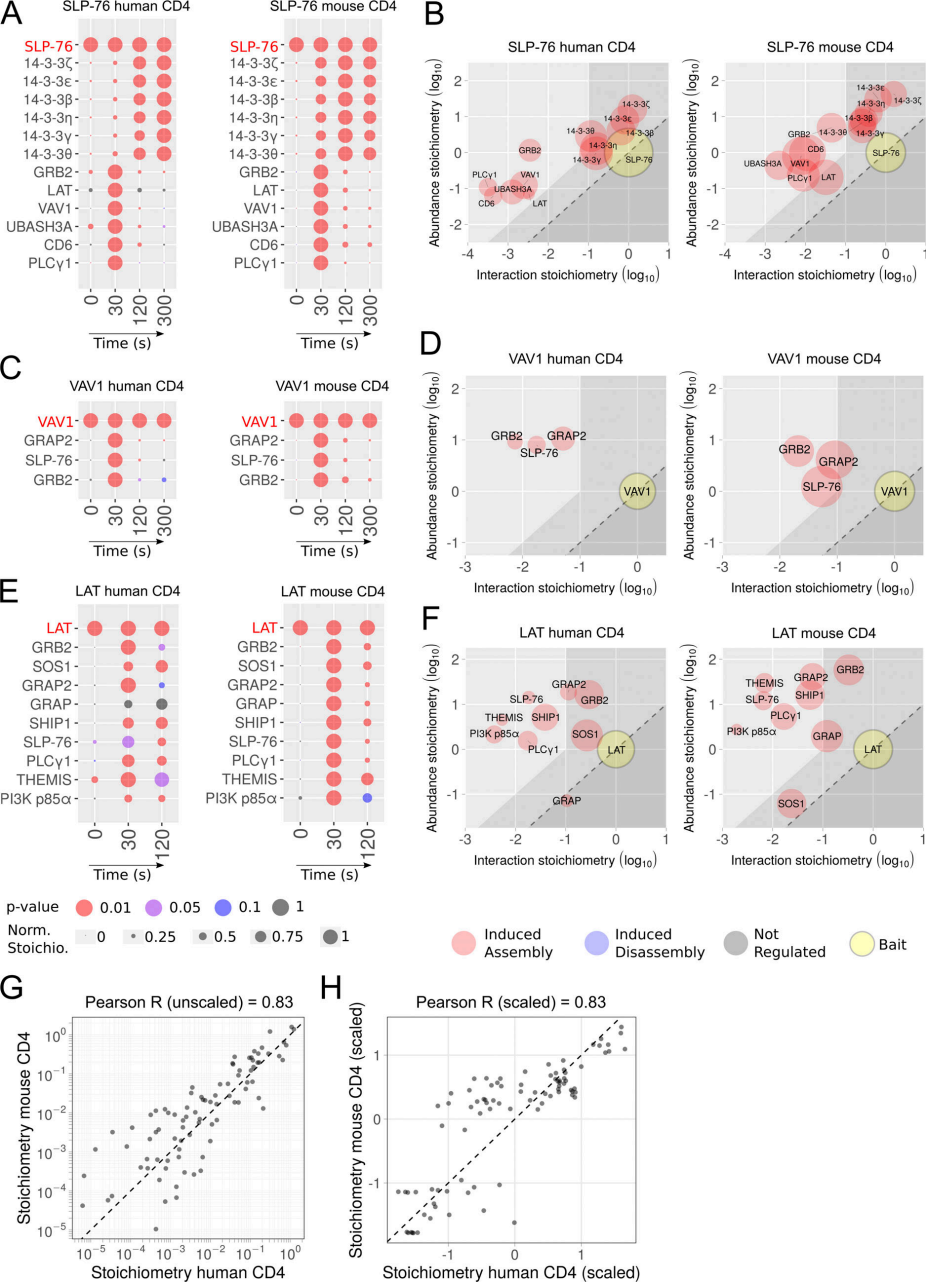
**Comparative analysis of the SLP-76, VAV1, and LAT interactomes of primary CD4^+^ T cells of human and mouse origins.** **(A)** Dot plots showing the interaction stoichiometry of SLP-76 in human and mouse CD4^+^ primary T cells over the course of TCR stimulation. For A, C, and E, see description in Fig. 2 C. In A–F, only the preys shared between CD4^+^ T cells of human and mouse origin are shown. **(B)** Stoichiometry plots of the SLP-76 interactome of CD4^+^ primary T cells of human and mouse origin. For B, D, and F, see description in Fig. 2 D. **(C)** Dot plots showing the interaction stoichiometry of VAV1 in human and mouse CD4^+^ primary T cells over the course of TCR-CD28 stimulation. **(D)** Stoichiometry plots of the VAV1 interactome of CD4^+^ primary T cells of human and mouse origin. **(E)** Dot plots showing the interaction stoichiometry of LAT in human and mouse CD4^+^ primary T cells over the course of TCR-CD28 stimulation. The role of the SUN domain–containing protein 2 (SUN2) that is found only in the LAT interactome of human CD4^+^ T cells remains to be elucidated. **(F)** Stoichiometry plots of the LAT interactome of CD4^+^ primary T cells of human and mouse origin. **(G)** The interaction stoichiometries of TCR-regulated interactions identified in both human and mouse CD4^+^ T cells and corresponding to the SLP76, VAV1, and LAT baits were compared across all conditions of stimulation. **(H)** Same comparison as in G using scaled (Z-score) interaction stoichiometries to compare variations of interaction stoichiometries relative to their means. Also shown in G and H are the Pearson correlation coefficient and a dashed line corresponding to equal stoichiometries (G) or scaled stoichiometries (H). Scaling was performed for each interaction by subtracting the mean and dividing it by the SD of log-transformed stoichiometries across all conditions of stimulation. Similar cutoff values (more than fivefold enrichment with a P value <0.05 in at least one of the five conditions of stimulation) were used to analyze the human and mouse datasets.

To determine whether mouse data “translate” to humans (Ernst and Carvunis, 2018; Wagar et al., 2018), we compared the SLP-76, VAV1, and LAT interactomes of human CD4^+^ T cells (this study) to their published mouse counterparts (Mori et al., 2021; Voisinne et al., 2019). These datasets were generated using saturating concentrations of stimulatory anti-CD3 antibodies and the same integrated AP-MS pipeline, to minimize technical variations. Among the 17 TCR-regulated SLP-76–prey interactions found in human CD4^+^ T cells, 13 interactions involving 14-3-3 ζ, ε, β, η, γ, and θ and GRB2, LAT, VAV1, UBASH3A, CD6, and PLCγ1 were conserved in mouse CD4^+^ T cells, whereas 4 interactions involving HLA-DQB1, NCK2, NAGK, and CCT3 were found only in human CD4^+^ T cells (Fig. 6 A). The conserved TCR-regulated SLP-76–prey interactions had similar dynamics, demonstrating that in both species, full-fledged SLP-76 signalosomes were already assembled 30 s after TCR activation and dismantled 120 s later. The three TCR-regulated VAV1–prey interactions found in primary human CD4^+^ T cells (GRAP2, SLP-76, and GRB2) were also present in mouse CD4^+^ T cells and displayed the same dynamics (Fig. 6 C). A trend toward interspecies conservation was also observed for VAV1–prey interaction stoichiometries (Fig. 6 D), as well as for LAT–prey interactions (see below). Accordingly, the stoichiometries of all the SLP-76–prey, VAV1–prey, and LAT–prey interactions that were shared between human and mouse CD4^+^ T cells showed a Pearson correlation coefficient of 0.83 (Fig. 6 G). Scaled (Z-score) interaction stoichiometries were also correlated (Pearson correlation coefficient of 0.83), indicating that the kinetics of interaction were similar between human and mouse CD4^+^ T cells (Fig. 6 H). Such correlation coefficients, however, were smaller than those observed when human CD4^+^ and CD8^+^ T cells were compared. It might reflect that the mouse SLP-76 and VAV-1 interactomes were obtained using SLP-76^OST^ and VAV-1^OST^ CD4^+^ T cells that were isolated from a gene-edited mouse and expanded in vitro for only 4 d in the presence of IL-2 (Voisinne et al., 2019), whereas their human counterparts were expanded for 34 d in vitro in the presence of IL-2 and leucoagglutinin (see Materials and methods).

### Comparison of the LAT interactomes of human and mouse CD4^+^ T cells

The human and mouse LAT interactomes were both established using long-term expanded CD4^+^ T cells (Fig. 4, E and F; Mori et al., 2021), permitting a more appropriate interspecies comparison. Among the 10 TCR-regulated LAT–prey interactions found in human CD4^+^ T cells, 9 were conserved in mouse CD4^+^ T cells, and they involved GRB2, the guanine nucleotide exchange factor SOS1, SLP-76, PLCγ1, GRAP2, the cytosolic adaptors GRAP and thymocyte-expressed molecule involved in selection (THEMIS), the phosphatidylinositol 3,4,5-trisphosphate 5-phosphatase 1 SHIP-1, and the phosphatidylinositol-3-kinase regulatory subunit α (also known as PI3-kinase subunit p85-α or PIK3 p85a; Fig. 6 E). Each of the 9 conserved LAT–prey interactions had similar dynamics, peaking 30–60 s after TCR stimulation. Mouse and human CD4^+^ T cells contained (within a factor of 2.3) similar numbers of LAT, GRB2, GRAP2, SHIP-1, PLCγ1, and SLP-76 molecules (Data S2; Mori et al., 2021). In contrast, long-term expanded human CD4^+^ T cells contained 50-fold more SOS1 molecules than long-term expanded mouse CD4^+^ T cells (Fig. 6 F and Data S2; Mori et al., 2021). As discussed below, this marked difference in SOS1 abundance did not translate, however, in the assembly of larger numbers of higher-order LAT-GRB2-SOS1-SLP-76-PLCγ1 signaling condensates in human CD4^+^ T cells.

### Assembly of higher-order LAT-GRB2-SOS1-SLP-76-PLCγ1 signaling condensates

**Figure 7.**
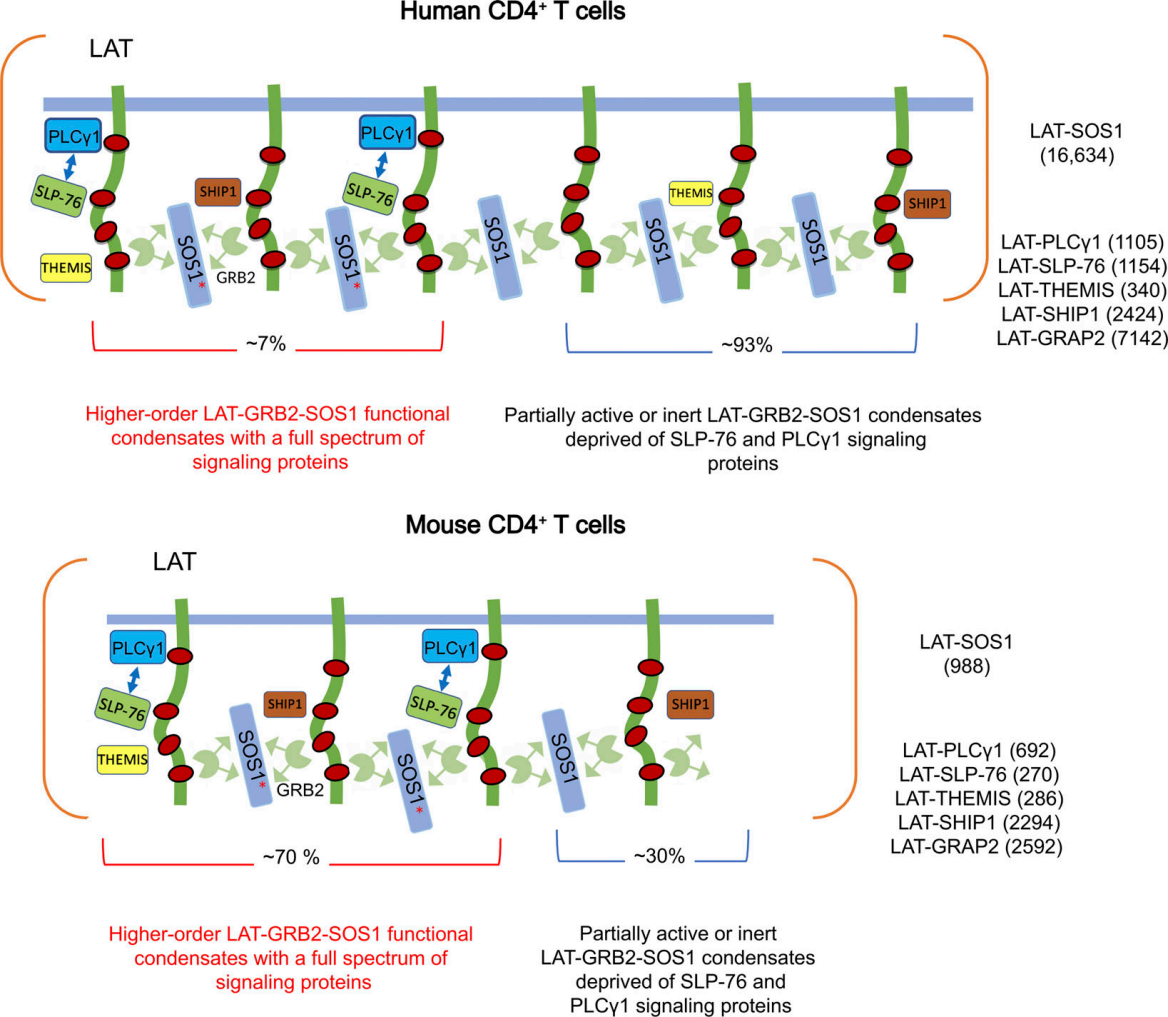
**Model summarizing the composition and stoichiometry of LAT condensates in human and mouse primary CD4^+^ T cells.** Model of the LAT-GRB2-SOS1–based condensates forming in human (top) and mouse (bottom) CD4^+^ T cells. The intrinsically disordered cytoplasmic tail of LAT is phosphorylated by ZAP-70 on at least three tyrosine residues (red dots) in response to TCR engagement, allowing interaction with a constellation of cytoplasmic proteins containing SH2 domains. These proteins also contain SH3 domains and proline-rich motifs, allowing them to establish multivalent interactions and enabling their assembly into scaffolds, and concomitant protein phase separation (Su et al., 2016). The client proteins recruited by LAT-GRB2-SOS1–based scaffold comprise SLP-76, THEMIS, SHIP-1, PIK3 p85a, GRAP2, and PLCγ1. They interact directly (PLCγ1 and GRAP2) or indirectly (THEMIS, SHIP-1, PIK3 p85a) with tyrosine phosphorylated LAT molecules. The maximal numbers of copies per T cell of LAT-SOS1, LAT-PLCγ1, LAT-SLP76, LAT-GRAP2, LAT-THEMIS, and LAT-SHIP-1 interactions reached 30 s after TCR engagement are specified in the right part of the human and mouse panels. These numbers likely correspond to a single conglomerate of individual LAT condensates (containing each a few hundreds of LAT molecules; McAffee et al., 2021 *Preprint*) that concatenated during the affinity purification process [see Discussion]).  Approximately 70% and 7% of the LAT-GRB2-SOS1 scaffold of mouse and human CD4^+^ T cells, respectively, are occupied by SLP-76 and PLCγ1 signaling clients, giving rise to comparable numbers of higher-order LAT-GRB2-SOS1-SLP-76-PLCγ1 signaling condensates capable of delivering full-fledged signals (see Results). SOS1 recruitment to the plasma membrane depends on its GRB2-dependent binding to phosphorylated LAT. This allows the autoinhibitory domains of SOS1 to interact with negatively charged membrane lipids, leading to structural rearrangements and subsequent release of autoinhibition. However, SOS1 full activity is contingent on prior activation of the the RAS exchange factor RAS-GRPP1 by the second messenger molecules diacylglycerol and calcium generated by active PLCγ1 molecules. It results in the production of RAS-GTP molecules that bind to an allosteric pocket on SOS1, increasing its catalytic activity up to 80-fold and allowing it to catalyzes nucleotide exchange on RAS molecules (Jun et al., 2013). Considering that RAS-GRP1 activity depends on PLCγ1 activity, fully active SOS1 molecules (red asterisk) are likely located within the higher-order LAT-GRB2-SOS1-SLP-76-PLCγ1 signaling condensates. In contrast, LAT-GRB2-THEMIS and LAT-GRB2-SHIP-1 ternary interactions do not rely on multivalent weak cooperative interactions as LAT, GRAP2, SLP-76, and PLCγ1 do, and are likely evenly distributed among the LAT-GRB2-SOS1 scaffold. The excess of LAT-GRAP2 interactions over LAT-SLP76 interactions in both mouse and human CD4^+^ T cells is likely due to the competition that exists between preformed GRAP2-SLP-76 binary complexes and the larger pool of free GRAP2 adaptors for binding to phosphorylated LAT molecules (Mori et al., 2021). For the sake of simplicity, RAS-GRP1, the GRAP2 adaptor bridging LAT and SLP-76 molecules, and the GRB2 adaptor bridging LAT and either THEMIS or SHIP-1 molecules are not represented. Some client proteins such as PLCγ1 can also contribute to phase separation (Zeng et al., 2021). SLP-76 has also been reported to contribute to oligomerization by binding to the adaptor molecule FYN-binding protein 1 (FYB; also known as ADAP; Coussens et al., 2013). However, we failed to detect FYB in the SLP-76 and LAT interactomes of both human and mouse primary T cells as well as VAMP7 in the LAT interactome (Data S1; Mori et al., 2021).

Upon phosphorylation, the three most carboxy-terminal tyrosine residues of LAT can bind to GRB2, and each of the LAT-bound GRB2 is capable of recruiting one SOS1 molecule to the plasma membrane. Considering that a single SOS1 molecule can associate with two GRB2 molecules, multivalent binding events ensue, leading to the abrupt oligomerization of LAT-GRB2-SOS1 complexes (Houtman et al., 2004, 2006; Nag et al., 2009; Kortum et al., 2013; McAffee et al., 2021 *Preprint*), and to protein phase separation (Fig. 7; Su et al., 2016). Protein phase separation induced by the LAT-GRB2-SOS1 scaffold stabilizes the recruitment of “client” proteins and organizes them into liquid-like condensates (also known as microclusters; Balagopalan et al., 2021). They facilitate downstream signaling events such as actin polymerization and activation of the RAS-MAPK-ERK, NFAT, and NF-κB signaling pathways (Ditlev et al., 2018; Ditlev et al., 2019; Huang et al., 2017; Su et al., 2016). The client proteins recruited by phosphorylated LAT molecules within LAT-GRB2-SOS1 scaffold comprise SLP-76, PLCγ1, THEMIS, SHIP-1, and PIK3 p85a (Fig. 4 E).

The maximal number of LAT-GRB2-SOS1 complexes assembling in human CD4^+^ T cells (16,634 copies per T cell) is 17-fold larger than that assembled in mouse CD4^+^ T cells (988 copies per T cell; Fig. 6 F, Data S1, and Data S2). Considering that human and mouse CD4^+^ T cells expressed comparable levels of LAT and GRB2 “building blocks” (Data S2; Mori et al., 2021), this 17-fold difference is likely accounted for by the presence in human CD4^+^ T cells of 50-fold more copies of SOS1 molecules compared with mouse CD4^+^ T cells. Provided that the extent of LAT-GRB2-SOS1 scaffold per T cell is commensurate with the concentration of LAT-GRB2-SOS1 complexes, the maximal cell surface occupied by LAT-GRB2-SOS1 scaffold in human CD4^+^ T cells is expected to be 17-fold larger than that in mouse CD4^+^ T cells. Comparable maximal numbers (within a factor of 4) of LAT-SLP-76 and LAT-PLCγ1 interactions were reached in both mouse and human T cells (Fig. 7 and Data S1). Considering that multivalent weak cooperative interactions are required to stabilize the LAT-SLP-76 and LAT-PLCγ1 interactions (Manna et al., 2018), the SLP-76 and PLCγ1 client proteins are likely not independently distributed across the LAT-GRB2-SOS1 scaffold but rather constitute higher-order LAT-GRB2-SOS1-SLP-76-PLCγ1 signaling condensates. Accordingly, by determining the ratio of the maximal numbers of LAT-PLCγ1 complexes (used as a proxy of higher-order LAT-GRB2-SOS1-SLP-76-PLCγ1 signaling condensates) to the maximal numbers of LAT-SOS1 complexes (used as a proxy of LAT-GRB2-SOS1 scaffold extent), it can be calculated that ∼70% and 7% of the LAT-GRB2-SOS1 scaffold of mouse and human CD4^+^ T cells, respectively, were associated with SLP-76 and PLCγ1 signaling clients and thus capable of delivering full-fledged signals (Fig. 7). Due to the larger LAT-GRB2-SOS1 scaffold found in human CD4^+^ T cells, it translated, however, in the assembly of a comparable maximum number of higher-order LAT-GRB2-SOS1-SLP-76-PLCγ1 signaling condensates in mouse (667 copies) and human (996 copies) CD4^+^ T cells (Fig. 7). Therefore, the signals delivered by the human and mouse proximal TCR signal transduction network following TCR engagement are likely of comparable magnitude.

## Discussion

We developed a gene-tagging platform that uses Cas9/sgRNA RNP nucleofection and nonviral HDR DNA templates and renders primary human CD4^+^ and CD8^+^ T cells rapidly amenable to AP-MS. It allowed us to define, at the systems level, the composition, dynamics, and stoichiometry of the human proximal TCR signal transduction network and chart its similarities and differences between human CD4^+^ and CD8^+^ T cells and across human and mouse species. Although humans and mice derive from a common mammalian ancestor and evolved independently for >90 million years, the composition and dynamics of the human and mouse proximal TCR signal transduction networks show a high degree of evolutionary conservation. The wealth of interactors assembling around the human and mouse SLP-76 orthologues upon TCR engagement support that view, and similar trends were observed for the smaller numbers of interactors detected in the LAT and VAV1 interactomes. Not only have the human and mouse documented orthologue baits kept similar interaction partners, but the dynamics of recruitment of those interaction partners over 300 s of TCR engagement and their interaction stoichiometries were also globally conserved between mouse and human.

Among the few mouse–human interspecies differences, we noted that only the human ZAP-70 molecules interacted with CD5 molecules after TCR engagement. The LAT–SOS1 interaction, which occurs via GRB2, constituted another notable exception to this interspecies conservation. Human CD4^+^ T cells contained 50-fold more copies of SOS1 molecules than mouse CD4^+^ T cells, and the maximal number of LAT-GRB2-SOS1 complexes assembling in human CD4^+^ T cells was 17-fold larger than that assembled in mouse CD4^+^ T cells. However, we showed that the presence of a wider LAT-GRB2-SOS1 scaffold in human CD4^+^ T cells was not accompanied by a commensurate increase in higher-order LAT-GRB2-SOS1-SLP-76-PLCγ1 signaling condensates. Therefore, since the assembly of higher-order and fully functional LAT condensates is more demanding than that of a LAT-GRB2-SOS1 scaffold devoid of SLP-76 and PLCγ1, the sole quantification of the extent of a LAT-GRB2-SOS1 scaffold present at the level of the T cell plasma membrane cannot be used to infer that it delivers full-fledged signals.

A hybrid live cell-supported membrane interface functionalized with peptide-MHC (pMHC) complexes was recently used to track the formation, duration, and movement of individual pMHC:TCR binary complexes while simultaneously monitoring LAT condensation in response to each pMHC:TCR binding event (McAffee et al., 2021 *Preprint*). It revealed that a single pMHC:TCR binding event is sufficient to trigger formation of a LAT condensate containing 258 ± 65  LAT molecules and endowed with a mean lifetime of a few tens of seconds (Britain and Weiner, 2021 *Preprint*; McAffee et al., 2021 *Preprint*). In the present study, we artificially cross-linked the TCR with saturating amounts of specific antibodies to initiate synchronized signaling in 100 x 10^6^ edited human T cells, which is essential to follow signaling dynamics by AP-MS. It thus markedly differs from the above experiments, which examine at single-cell level the low antigen end of the spectrum to measure how sparsely distributed pMHC:TCR binding events trigger individual LAT condensates. Intriguingly, these two orthogonal approaches concur to show that LAT condensates have a mean lifetime of a few tens of seconds that results in part from the action of 14-3-3 phosphoserine-phosphothreonine-binding proteins (this study and Mori et al., 2021), and prevents undesirable feed-forward loop. 

Although our approach provides an unprecedented, systems-level picture of the human proximal TCR signal transduction network, it presents a few limitations that must be noted. Our current AP-MS pipeline required to analyze 100 × 10^6^ edited human T cells per time point and therefore provides an averaged picture of the hundreds of individual signalosomes assembling in each analyzed T cell. Addressing the possibility that distinct signalosome isoforms exist within the same T cell will thus require the use of single-molecule localization microscopy and hybrid live-cell–supported lipid bilayer models (Lin et al., 2019). When the long-term expanded human T cells were collected for AP-MS, they were at the end of an expansion cycle and stopped proliferating. Therefore, our results reflect the behavior of the proximal TCR signaling network of human primary T cell lymphoblasts that were expanded and rested down before TCR stimulation. The present pilot study also used CD3 plus CD28 cross-linkage, a less physiological stimulation condition than that relying on interaction of T cells with APCs. Tagging antigen-specific human T cell clones should permit stimulation with APCs or tumor cells in the case they derived from tumor-infiltrating lymphocytes.

In conclusion, our study provides the most comprehensive systems-level analysis yet on the composition, stoichiometry, and dynamics of the proximal TCR signal-transduction network in primary human T cells. Our study revealed that the proximal TCR signal transduction network has a high degree of qualitative and quantitative conservation across human and mouse T cells. Independently of evolutionary considerations, and as illustrated with an inhibitor of the LCK PTK, the possibility to render primary human T cells rapidly amenable to AP-MS permits us to precisely define whether a drug acts upstream or downstream of a given signalosome of the human TCR signal transduction network. Therefore, the fast-track system-level analysis of human T cell signaling pathways described here should be favored to optimize CAR T cell signaling and evaluate, at the systems level, the mechanisms of action and possible side effects of drugs targeting human T cell activation before translating them into the clinic.

## Materials and methods

### Primary human T cells and ethics approval

Human peripheral blood samples were obtained from anonymous healthy adult volunteers after informed consent in accordance with the local Research Ethics Committee of the Etablissement Français du Sang (Etablissement Français du Sang Nantes, Pays de la Loire, agreement PLER NTS 2018-21). All the reported T cell editing experiments were performed on PBMCs that originated from a single 48-yr-old healthy male donor to avoid potential variations resulting from the use of different blood donors.

### Design of sgRNA for editing the *ZAP70*,* LAT*,* LCP2*, and *VAV1* genes of primary human CD4^+^ and CD8^+^ T cells

sgRNAs of high cutting efficiency were designed using the Crispor algorithm (Haeussler et al., 2016) to introduce a double-strand break (DSB) close to the STOP codon of the *ZAP70*,* LAT*,* LCP2*, and *VAV1* genes. sgRNA sequences (Table S1) were modified with 2′-*O*-methyl 3′-phosphorothioate in the first and last three nucleotides (Hendel et al., 2015) and were purchased from Integrated DNA Technologies.

### Design of DNA HDR templates for editing the *ZAP70*,* LAT*,* LCP2*, and *VAV1* genes of primary human CD4^+^ and CD8^+^ T cells

For each bait, an 845-bp-long dsDNA HDR template was designed and purchased from Integrated DNA Technologies in a plasmid vector. Each HDR DNA template contained (1) a 100-bp-long sequence homologous to the sequence flanking the 5′ end of the intended DSB (5′ homology arm); (2) a 81-bp-long OST tag sequence (Junttila et al., 2005) flanked on both sides by a Gly-Ser-Gly spacer; (3) a 57-bp-long sequence coding for the self-cleaving P2A peptide (Kim et al., 2011); (4) a 489-bp-long sequence coding for the mouse CD90.1 protein; and (5) a 100-bp-long sequence homologous to the sequence flanking the 3′ end of the DSB (3′ homology arm). The sequences of the HDR DNA templates used to edit the *ZAP70*,* LAT*,* LCP2*, and *VAV1* genes are listed in Table S2. The HDR DNA template present in each plasmid vector was amplified by PCR and purified with QIAquick PCR purification kit (Qiagen) before nucleofection. To prevent CRISPR-Cas9 cleavage of the edited alleles, a silent mutation destroying the PAM sequence present in the genomic DNA was introduced into the HDR DNA templates.

### Knock-in of OST-P2A-CD90.1–coding HDR templates and selection of edited CD4^+^ and CD8^+^ T cells

PBMCs were thawed and stimulated with Dynabeads human T-activator CD3-CD28 (11131D; Gibco) at a ratio of three beads for one cell in the presence of IL-2 (30 IU/ml). After 48 h, beads were removed, and the cells were electroporated using a P3 primary cell 4D-Nucleofector X kit L (V4XP-3024; Lonza) and the program EO-115. Before electroporation, RNP complexes were formed by incubating equal volumes of sgRNA (44 µM) and Alt-R S.p. HiFi Cas9 nuclease V3 (36 µM; 1081061; Integrated DNA Technologies) at room temperature for 20 min according to the manufacturer’s instructions. 1.5–3.0 × 10^6^ activated T cells were electroporated with either 10 µl RNP plus 10 µg of HDR DNA template (edited T cells) or Cas9 alone (control T cells). Cells were analyzed by flow cytometry for CD90.1 expression 4 d after transfection using anti–CD4-APC (555349; BD Biosciences), anti–CD8-FITC (555366; BD Biosciences), and anti–CD90.1-PE (202524; BioLegend) antibodies. Edited T cells were sorted into CD4^+^CD90-1^+^ and CD8^+^CD90.1^+^ subpopulations 5 d after transfection using a FACS Aria cell sorter (BD Biosciences). CD4^+^CD90.1^−^ cells and CD8^+^CD90.1^−^ control T cells were sorted in parallel and used as negative controls.

### Expansion of properly edited CD4^+^ and CD8^+^ T cells

CD90.1^+^CD4^+^ and CD90.1^+^CD8^+^ sorted T cells were put back in culture for 9 d in the presence of 300 IU/ml human IL-2 (Novartis Proleukin). T cells were then distributed in 96-well plates (10^3^ cells per well) in 200 µl RPMI 1640 (31870-025; Gibco) supplemented with irradiated (35 Gy) pooled allogenic feeder cells (corresponding to 10^7^ PBMCs from a pool of three donors and 10^6^ cells corresponding to a 1-to-1 mix of two B-lymphoblastoid cell lines established from distinct donors), 1 µg/ml leukoagglutinin (PHA-L; 4144-5MG; Sigma-Aldrich), 7% pooled human serum, GlutaMAX (35050-038; Gibco), penicillin-streptomycin (15140-122; Gibco), and IL-2 (300 IU/ml) as described (Vivien et al., 2018). After 7 d of culture in 96-well plates, T cells were expanded in RPMI 1640 supplemented with 7% pooled human serum, GlutaMAX, penicillin/streptomycin, and 300 IU/ml human IL-2 for an additional 10 d, a time point when T cells stopped proliferating and reached numbers appropriate for AP-MS analysis. Before use in AP-MS analysis, T cells were analyzed for the expression of mouse CD90.1, and human CD3 and CD28 using anti–CD90.1-BV605 (740373; BD Biosciences), anti–CD3-APC (17-0037-41; Invitrogen), and anti–CD28-FITC (302906; BD Biosciences) antibodies. Control T cells were grown in parallel under the same conditions.

### TCR Vβ repertoire analysis

The TCR Vβ repertoire diversity of the expanded T cells was determined using a panel of 24 TCR Vβ-specific antibodies (Beta Mark TCR Vβ Repertoire Kit; IM3497; Beckman Coulter). After staining, cells were analyzed with a C6 Accuri Flow Cytometer (BD Biosciences).

### Stimulation and lysis of expanded human CD4^+^ and CD8^+^ T cells before AP-MS analysis

Expanded CD4^+^CD90-1^+^ and CD8^+^CD90.1^+^ T cells (100 × 10^6^) were incubated with anti-CD3 (0.2 µg per 10^6^ cells; OKT3; 317347; BioLegend) and anti-CD28 (0.2 µg per 10^6^ cells; CD28.2; 302943; BioLegend) for 15 min on ice, followed by one round of washing at 4°C. Cells were then incubated at 37°C for 5 min and stimulated at 37°C by cross-linking the bound anti-CD3 and anti-CD28 antibodies with purified rabbit anti-mouse IgG F(ab′)_2 _(0.4 µg per 10^6^ cells; Jackson ImmunoResearch) for 30, 60, 120, and 300 s, or left unstimulated. The stimulation was stopped by the addition of twice-concentrated lysis buffer (100 mM Tris, pH 7.5, 270 mM NaCl, 1 mM EDTA, 20% glycerol, 0.4% *n*-dodecyl-β-D-maltoside) supplemented with protease and phosphatase inhibitors. After 10 min of incubation on ice, cell lysates were centrifuged at 21,000 *g* for 5 min at 4°C. Postnuclear cell lysates were used for immunoblot analysis or affinity purification. Three independent replicates were performed over two to three consecutive days, and control T cells were processed in parallel under the same conditions.

### Dasatinib treatment

Dasatinib (SML2589-50MG; Sigma-Aldrich) was dissolved in DMSO to a concentration of 4 mM and diluted in RPMI 1640 to a final concentration of 100 nM. SLP-76^OST^ CD4^+^ T cells and control cells were expanded as previously described and preincubated for 45 min at 37°C with Dasatinib (100 nM) or the DMSO vehicle alone. Dasatinib (100 nM) or DMSO was also kept during all subsequent steps until cell lysis.

### Affinity purification of OST-tagged protein complexes

Equal amounts of postnuclear cell lysates were incubated with prewashed Strep-Tactin Sepharose beads (IBA) for 1.5 h at 4°C on a rotary wheel. Beads were then washed three times with 1 ml of lysis buffer containing protease and phosphatase inhibitors in the absence of detergent and further washed twice with 1 ml of lysis buffer in the absence of protease and phosphatase inhibitors and detergent. Proteins were eluted from the Strep-Tactin Sepharose beads with 2.5 mM D-biotin, a ligand that binds to Strep-Tactin with a higher affinity than the OST sequence does.

### Western blot analysis of OST-tagged human T cells

Affinity-purified samples and whole-cell lysates were loaded on 8–10% SDS-PAGE gel and subsequently analyzed by immunoblot with specific antibodies. The following antibodies were used for immunoblot analysis: anti–ZAP-70 (2705; Cell Signaling Technology), anti-LAT (9166; Cell Signaling Technology), anti–SLP-76 (4958; Cell Signaling Technology), anti-VAV1 (2502; Cell Signaling Technology), and anti-phosphotyrosine (4G10; Millipore).

### Tandem MS analysis

Following affinity purification, protein samples were air dried in a Speed-Vac concentrator and either reconstituted in Laemmli buffer and processed by SDS-PAGE and trypsin in-gel digestion as previously described (Voisinne et al., 2019) or reconstituted in 5% SDS/50 mM ammonium bicarbonate and processed for trypsin digestion using a S-trap micro device (Protifi) according to the manufacturer instructions. Tryptic peptides were resuspended in 17 µl of 2% acetonitrile and 0.05% trifluoroacetic acid and analyzed by nano-LC coupled to tandem MS, using an UltiMate 3000 system (NCS-3500RS Nano/Cap System; Thermo Fisher Scientific) coupled to an Orbitrap Q Exactive mass spectrometer (model Q Exactive Plus; Thermo Fisher Scientific). 5 µl of each sample was loaded on a C18 precolumn (300 µm inner diameter × 5 mm; Thermo Fisher Scientific) in a solvent made of 2% acetonitrile and 0.05% trifluoroacetic acid, at a flow rate of 20 µl/min. After 5 min of desalting, the precolumn was switched online with the analytical C18 column (75 µm inner diameter × 50 cm; Acclaim PepMap C18, 2 µM; Thermo Fisher Scientific; or in-house packed with 3 µm Reprosil C18) equilibrated in 95% solvent A (5% acetonitrile, 0.2% formic acid) and 5% solvent B (80% acetonitrile, 0.2% formic acid). Peptides were eluted using a 5–50% gradient of solvent B over 60 min at a flow rate of 300 nl/min. The mass spectrometer was operated in data-dependent acquisition mode with Xcalibur software. MS survey scans were acquired with a resolution of 70,000 and an automatic gain control target of 3e6. The 10 most intense ions were selected for fragmentation by high-energy collision–induced dissociation, and the resulting fragments were analyzed at a resolution of 17500, using an automatic gain control target of 1e5 and a maximum fill time of 50 ms. Dynamic exclusion was used within 30 s to prevent repetitive selection of the same peptide.

### Protein identification and quantification for interaction proteomics

Raw MS files were processed with MaxQuant software (v1.5.2.8) for database search with the Andromeda search engine and quantitative analysis. Data were searched against *Homo sapiens* entries of the UniProt KB protein database (release UniProtKB/Swiss-Prot+TrEMBL 2019_09; 195,349 entries including isoforms), plus the One-Strep-tag peptide sequence and the set of common contaminants provided by MaxQuant. Carbamidomethylation of cysteines was set as a fixed modification, whereas oxidation of methionine, protein N-terminal acetylation, and phosphorylation of serine, threonine, and tyrosine were set as variable modifications. Specificity of trypsin digestion was set for cleavage after K or R, and two missed trypsin cleavage sites were allowed. The precursor mass tolerance was set to 20 ppm for the first search and 4.5 ppm for the main Andromeda database search. The mass tolerance in tandem MS mode was set to 0.5 dalton. Minimum peptide length was set to 7 amino acids, and minimum number of unique or razor peptides was set to 1 for validation. The I = L option of MaxQuant was enabled to avoid erroneous assignation of undistinguishable peptides belonging to very homologous proteins. Andromeda results were validated by the target decoy approach using a reverse database, with a false discovery rate set at 1% at both peptide sequence match and protein level. For label-free relative quantification of the samples, the match between runs option of MaxQuant was enabled with a match time window of 1 min, to allow cross-assignment of MS features detected in the different runs, after alignment of the runs with a time window of 20 min. Protein quantification was based on unique and razor peptides. The minimum ratio count was set to 1 for calculation of label-free quantification, and computation of the intensity-based absolute protein quantification (iBAQ) metric was also enabled.

### Data processing and identification of specific interactors

From the “proteinGroups.txt” files generated by MaxQuant with the options described above, protein groups with negative identification scores were filtered, as well as proteins identified as contaminants. When protein groups corresponded to the same gene name, protein intensities in a given sample were summed over the redundant protein groups. Protein intensities were normalized across all samples by the median intensity. Normalized intensities corresponding to different technical replicates were averaged (geometric mean), and missing values were replaced after estimating background binding from WT intensities. For each bait and each condition of stimulation, we used a two-tailed Welch’s *t* test to compare normalized log-transformed protein intensities detected in OST-tagged samples across all biological replicates to control sample intensities pooled from all conditions of stimulation. To avoid spurious identification of interactors due to missing value imputation, we repeated 10 times the “missing value imputation followed by a two-tailed Welch’s *t* test” process and estimated fold-changes and P values as their respective average (geometric mean) across all 10 tests. Specific interactors were identified as preys showing a greater than fivefold enrichment with a P value <0.05 in at least one condition of stimulation.

### Calculation of interaction stoichiometries

For a given condition of stimulation (represented by the time of stimulation *t*, with *t* = 0 s corresponding to the nonstimulated condition), the stoichiometry of the interaction between a prey *x* and a given bait (denoted *bait*<*x)* was computed using

$$S_{bait<x}(t)=I_{OST,x}(t)/I_{OST,bait}(t)\times N_{pep,bait}/N_{pep,x},$$

where *I_OST,x_* corresponds to the normalized intensity of protein *x* in OST-tagged samples and stimulation time *t* averaged (geometric mean) across all biological replicates, and *N_pep_* corresponds to the number of tryptic peptides theoretically observables as estimated from iBAQ values. We also computed stoichiometries independently for each biological replicate and used those values to quantify the regulation of bait–prey association following TCR engagement. For a given condition of stimulation, log-transformed stoichiometries were compared with that of the nonstimulated condition using a two-tailed Welch’s *t* test. Prey–bait pairs whose interaction stoichiometry changed at least twofold with a P value <0.1 in at least one condition of stimulation as compared with the nonstimulated condition are defined as significantly regulated upon TCR stimulation.

### MS determination of the sequence straddling the C-terminal end of each bait protein and the OST tag sequence

To check that each bait protein was correctly expressed along with the OST tag sequence, raw MS files were analyzed using the Mascot search engine (Matrix Science) to compare MS/MS spectra against a version of the human UniProt KB reference proteome containing the expected sequence of each bait following CRISPR/Cas9-based edition. The sequence of the peptides straddling the C-terminal end of each bait protein and the OST tag sequence were validated at 1% false discovery rate by target-decoy search using Proline software (Bouyssié et al., 2020).

### High-resolution MS characterization of the proteome of long-term expanded CD4^+^ and CD8^+^ T cells

For proteome analysis, cell pellets corresponding to 5 × 10^6^ expanded CD4^+^ and CD8^+^ T cells were incubated with 150 µl of lysis buffer containing 50 mM Tris, pH 7.5, 0.5 mM EDTA, 135 mM NaCl, and 1% SDS for 10 min on ice and subjected to sonication with a Bioruptor ultrasonicator. An aliquot of 100 µg of total protein from each sample was digested by trypsin on a S-trap mini device (Protifi). Tryptic peptides were dried and resuspended in 125 µl of 2% acetonitrile and 0.05% trifluoroacetic acid. 5 µl of each sample were analyzed with by nano-LC-MS on an Orbitrap Q Exactive mass spectrometer (Q Exactive HFX; Thermo Fisher Scientific) with the same LC configuration as described above, but with a longer separation gradient (10–45% of solvent B over 120 min). Raw MS files were processed with MaxQuant as described above, except that phosphorylation was not included as a variable modification for the database search.

### Calculation of protein copy numbers per T cell

Analysis of the proteome of long-term expanded human CD4^+^ and CD8^+^ T cells identified 5,596 protein groups. Protein entries from the MaxQuant “proteinGroups.txt” output with a negative identification score or with a null total count of MS/MS identifications were filtered, as well as entries from reverse and contaminant databases. Cellular protein abundances were determined from raw intensities with the protein ruler methodology (Wiśniewski et al., 2014), using the following relationship: protein copies per cell = (protein MS signal × *N_A_* × DNA mass)/(*M* × histone MS signal), where *N_A_* is Avogadro’s constant, *M* is the molar mass of the protein, and the DNA mass of a diploid human cell is estimated to be 6.6 pg. Cellular protein abundances were averaged (geometric mean) sequentially over technical and biological replicates. Overall, the cellular protein abundance could be estimated for 5,221 and 4,976 protein groups in human CD4^+^ and CD8^+^ T cells, respectively. The cellular protein abundance of short-term expanded WT mouse CD4^+^ T cells is from Voisinne et al. (2019). The cellular protein abundance of long-term expanded WT mouse CD4^+^ T cells is from Mori et al. (2021).

### Quantification of posttranslational modifications in SLP-76^OST^ AP-MS samples

Phosphorylation site quantification was performed using the intensities of the “Phospho (STY).txt” file from the MaxQuant analysis of SLP-76^OST^ AP-MS samples. Phosphorylation intensities for sites identified on SLP-76 were normalized by the intensity of the SLP-76 protein group extracted from the “proteinGroups.txt” file from the same MaxQuant analysis. Normalized phosphorylation intensities were then averaged (geometric mean) across technical replicates.

### Online supplemental material

Fig. S1 shows the gating strategy used to assess CD90.1, CD3, CD4, and CD28 expression and TCR Vβ repertoire diversity in control and SLP-76^OST^–edited CD4^+^ T cells that were both expanded for 34 d. Fig. S2 shows the normalized bait protein intensities in AP-MS samples corresponding to CD4^+^ and CD8^+^ T cells from OST-tagged and control samples. Fig. S3 shows the sequence of the bait peptides straddling the C-terminal end of the bait protein and the OST tag as determined by AP-MS. Fig. S4 compares the protein abundance between CD4^+^ and CD8^+^ primary human T cells. Table S1 shows the sequence of the sgRNA. Table S2 shows the sequence of the dsDNA HDR templates. Table S3 shows the sequence of the peptide straddling the C-terminal end of each bait protein and the OST tag in edited CD4^+^ and CD8^+^ T cells. Data S1 lists the bait–prey interactions identified in the SLP-76, VAV1, ZAP-70, and LAT interactomes of CRISPR/Cas9-edited and expanded human primary CD4^+^ and CD8^+^ T cells. Data S2 lists the proteome of expanded primary CD4^+^ and CD8^+^ human T cells. Data S3 lists the bait–prey interactions identified in the SLP-76 interactomes of human primary CD4^+^ T cells in presence or absence of dasatinib treatment.

## Supplementary Material

Table S1 lists sgRNA sequences.

Table S2 lists HDR dsDNA template sequences.

Table S3 lists sequences of bait peptides.

Data S1 lists the bait–prey interactions identified in the SLP-76, VAV1, ZAP-70, and LAT interactomes of CRISPR/Cas9-edited and expanded human primary CD4^+^ and CD8^+^ T cells.

Data S2 lists the proteome of expanded primary CD4^+^ and CD8^+^ human T cells.

Data S3 lists the bait–prey interactions identified in the SLP-76 interactomes of human primary CD4^+^ T cells in presence or absence of dasatinib treatment.

## Data Availability

The MS proteomics data have been deposited to the ProteomeXchange Consortium via the Proteomics Identifications Database partner repository (https://www.ebi.ac.uk/pride) with the dataset identifiers: PXD024820 (LAT, SLP76, VAV1, and ZAP70 interactomes of expanded human CD4^+^ and CD8^+^ T cells), PXD025174 (proteomes of long-term expanded human CD4^+^ and CD8^+^ T cells), and PXD028939 (SLP-76 interactomes of expanded human CD4^+^ T cells in presence or absence of dasatinib treatment).
