# Supplementary material for: Systems-level conservation of the proximal TCR signaling network of mice and humans
Source: J Exp Med. 2022 Jan 21;219(2):e20211295. doi: 10.1084/jem.20211295 (PMC8789201; doi:10.1084/jem.20211295)
Supplement: Table S1 — lists sgRNA sequences. [file JEM_20211295_TableS1.docx]

Table S1. sgRNA sequences

| Gene | sgRNA target sequence (5′ to 3′) |
| --- | --- |
| ***LCP2*** | GCACATTAACGCATGCTGCA |
| ***ZAP70*** | CCCCTGGGCAGCGGGAGCTC |
| ***LAT*** | AATCTGCAGGAGCTGAACTG |
| ***VAV1*** | GTCTCTCTGCCAAGGCACCA |
