# Supplementary material for: Systems-level conservation of the proximal TCR signaling network of mice and humans
Source: J Exp Med. 2022 Jan 21;219(2):e20211295. doi: 10.1084/jem.20211295 (PMC8789201; doi:10.1084/jem.20211295)
Supplement: Table S2 — lists HDR dsDNA template sequences. [file JEM_20211295_TableS2.docx]

Table S2. **HDR dsDNA templates sequences**

| **Gene** | **Sequence** |
| --- | --- |
| ***LCP2*** | tattattgactacttcaggaaaatgccacttctgctcattgatgggaaaaaccgaggttccagataccagtgtactctaacacacgctgctggatatccaggaagcggatggagccacccgcagttcgagaaaggtggaggttccggaggtggaagtggcgggagctggtcacatccacaatttgaaaagggaagcggagctactaacttcagcctgctgaagcaggctggagacgtggaggagaaccctggacctatgaacccagccatcagcgtcgctctcctgctctcagtcttgcaggtgtcccgagggcagaaggtgaccagcctgacagcctgcctggtgaaccaaaaccttcgcctggactgccgccatgagaataacaccaaggataactccatccagcatgagttcagcctgacccgagagaagaggaagcacgtgctctcaggcaccctcgggatacccgagcacacgtaccgctcccgcgtcaccctctccaaccagccctatatcaaggtccttaccctagccaacttcaccaccaaggatgagggcgactacttttgtgagcttcgagtctcgggcgcgaatcccatgagctccaataaaagtatcagtgtgtatagagacaaactggtcaagtgtggcggcataagcctgctggttcagaacacatcctggatgctgctgctgctgctttccctctccctcctccaagccctggacttcatttctctgtagcaagttatagccgagcaaatgaaccgtcctcctgcctctgttgccaacacgagatcaatcagccttggtcaatggacaaacacttaggactgaactgaac |
| ***ZAP70*** | cctgaccgtggagcagcgcatgcgagcctgttactacagcctggccagcaaggtggaagggcccccaggcagcacacagaaggctgaggctgcctgtgccggaagcggatggagccacccgcagttcgagaaaggtggaggttccggaggtggaagtggcgggagctggtcacatccacaatttgaaaagggaagcggagctactaacttcagcctgctgaagcaggctggagacgtggaggagaaccctggacctatgaacccagccatcagcgtcgctctcctgctctcagtcttgcaggtgtcccgagggcagaaggtgaccagcctgacagcctgcctggtgaaccaaaaccttcgcctggactgccgccatgagaataacaccaaggataactccatccagcatgagttcagcctgacccgagagaagaggaagcacgtgctctcaggcaccctcgggatacccgagcacacgtaccgctcccgcgtcaccctctccaaccagccctatatcaaggtccttaccctagccaacttcaccaccaaggatgagggcgactacttttgtgagcttcgagtctcgggcgcgaatcccatgagctccaataaaagtatcagtgtgtatagagacaaactggtcaagtgtggcggcataagcctgctggttcagaacacatcctggatgctgctgctgctgctttccctctccctcctccaagccctggacttcatttctctgtaggctcccgctgcacaggggagccctccacgccggctcttccccaccctcagccccaccccaggtcctgcagtctggctgagccctgcttggttgtctc |
| ***LAT*** | gaccaacctcccctcccttacagccgccctgagttcccaggaggcagaggaagtggaggaagagggggctccagattacgagaatctgcaagagctgaacggaagcggatggagccacccgcagttcgagaaaggtggaggttccggaggtggaagtggcgggagctggtcacatccacaatttgaaaagggaagcggagctactaacttcagcctgctgaagcaggctggagacgtggaggagaaccctggacctatgaacccagccatcagcgtcgctctcctgctctcagtcttgcaggtgtcccgagggcagaaggtgaccagcctgacagcctgcctggtgaaccaaaaccttcgcctggactgccgccatgagaataacaccaaggataactccatccagcatgagttcagcctgacccgagagaagaggaagcacgtgctctcaggcaccctcgggatacccgagcacacgtaccgctcccgcgtcaccctctccaaccagccctatatcaaggtccttaccctagccaacttcaccaccaaggatgagggcgactacttttgtgagcttcgagtctcgggcgcgaatcccatgagctccaataaaagtatcagtgtgtatagagacaaactggtcaagtgtggcggcataagcctgctggttcagaacacatcctggatgctgctgctgctgctttccctctccctcctccaagccctggacttcatttctctgtgagggcatggtgagaggcctgccctgtccccaccctgccctgggcccacaggctctactccttccctccaggacccccttgcccaaccctacctctggcttg |
| ***VAV1*** | taggaggatgtgcagaggttgcactgatgaactcctcgtctgtttccaggttggctggttccctgccaactacgtggaagaagattattctgaatactgcggaagcggatggagccacccgcagttcgagaaaggtggaggttccggaggtggaagtggcgggagctggtcacatccacaatttgaaaagggaagcggagctactaacttcagcctgctgaagcaggctggagacgtggaggagaaccctggacctatgaacccagccatcagcgtcgctctcctgctctcagtcttgcaggtgtcccgagggcagaaggtgaccagcctgacagcctgcctggtgaaccaaaaccttcgcctggactgccgccatgagaataacaccaaggataactccatccagcatgagttcagcctgacccgagagaagaggaagcacgtgctctcaggcaccctcgggatacccgagcacacgtaccgctcccgcgtcaccctctccaaccagccctatatcaaggtccttaccctagccaacttcaccaccaaggatgagggcgactacttttgtgagcttcgagtctcgggcgcgaatcccatgagctccaataaaagtatcagtgtgtatagagacaaactggtcaagtgtggcggcataagcctgctggttcagaacacatcctggatgctgctgctgctgctttccctctccctcctccaagccctggacttcatttctctgtgagcactggtgccttggcagagagacgagaaactccaggctctgagcccggcgtgggcaggcagcggagccaggggctgtgacagctcccggcgggtggaga |

**Color code**

5’ homology arm sequence

3’ homology arm sequence

Gly-Ser-Gly spacer sequence

OST tag sequence

P2A peptide sequence

CD90.1 protein sequence

Stop codon sequence
