# Supplementary material for: Systems-level conservation of the proximal TCR signaling network of mice and humans
Source: J Exp Med. 2022 Jan 21;219(2):e20211295. doi: 10.1084/jem.20211295 (PMC8789201; doi:10.1084/jem.20211295)
Supplement: Table S3 — lists sequences of bait peptides. [file JEM_20211295_TableS3.docx]

Table S3. Sequences of the bait peptides straddling the C-terminal end of the bait protein and the OST tag, as determined by AP-MS

| Cells | Bait | Overlapping peptide sequence | Mascot psm score | Peptide length | Is protein set validated |
| --- | --- | --- | --- | --- | --- |
| **CD4^+^ T cells** | **SLP-76** | YQCTLTHAAGYPGSGWSHPQFEK | 93,44 | 23 | True |
|  | **ZAP-70** | AEAACAGSGWSHPQFEK | 102,49 | 17 | True |
|  | **LAT** | TEPAALSSQEAEEVEEEGAPDYENLQELNGSGWSHPQFEK | 54,12 | 40 | True |
|  | **VAV1** | VGWFPANYVEEDYSEYCGSGWSHPQFEK | 68,07 | 28 | True |
| **CD8^+^ T cells** | **SLP-76** | YQCTLTHAAGYPGSGWSHPQFEK | 91,77 | 23 | True |
|  | **ZAP-70** | AEAACAGSGWSHPQFEK | 113,77 | 17 | True |
|  | **VAV1** | VGWFPANYVEEDYSEYCGSGWSHPQFEK | 69,53 | 28 | True |
